# A Rho-based reaction-diffusion system governs cell wall patterning in metaxylem vessels

**DOI:** 10.1038/s41598-018-29543-y

**Published:** 2018-08-01

**Authors:** Yoshinobu Nagashima, Satoru Tsugawa, Atsushi Mochizuki, Takema Sasaki, Hiroo Fukuda, Yoshihisa Oda

**Affiliations:** 1 Department of Biological Sciences, Graduate School of Science, The University of Tokyo, 7-3-1 Hongo, Bunkyo-ku, Tokyo, 113-0033 Japan; 2 Center for Frontier Research, National Institute of Genetics, 1111 Yata, Mishima, Shizuoka, 411-8540 Japan; 3 Theoretical Biology Laboratory, RIKEN, 2-1 Hirosawa, Wako, Saitama, 351-0198 Japan; 4 Laboratory of Mathematical Biology, Institute for Frontier Life and Medical Sciences, Kyoto University, 53 Shogoin Kawahara-cho, Sakyo-ku, Kyoto, 606-8507 Japan; 5 CREST, Japan Science and Technology Agency, 4-1-8 Honcho, Kawaguchi, Saitama, 332-0012 Japan; 6 Department of Genetics, SOKENDAI (The Graduate University for Advanced Studies), 1111 Yata, Mishima, Shizuoka, 411-8540 Japan

## Abstract

Rho GTPases play crucial roles in cell polarity and pattern formation. ROPs, Rho of plant GTPases, are widely involved in cell wall patterning in plants, yet the molecular mechanism underlying their action remains unknown. Arabidopsis ROP11 is locally activated to form plasma membrane domains, which direct formation of cell wall pits in metaxylem vessel cells through interaction with cortical microtubules. Here, we show that the pattern formation of cell wall pits is governed by ROP activation *via* a reaction-diffusion mechanism. Genetic analysis and reconstructive assays revealed that *ROPGEF4/7* and *ROPGAP3/4*, which encode ROP activators and inactivators, respectively, regulated the formation of ROP-activated domains; these in turn determined the pattern of cell wall pits. Mathematical modelling showed that ROP-activation cycle generated ROP domains by reaction-diffusion mechanism. The model predicted that a positive feedback and slow diffusion of ROP11-ROPGEF4 complex were required to generate ROP-activated domains. ROPGEF4 formed a dimer that interacted with activated ROP11 *in vivo*, which could provide positive feedback for ROP activation. ROPGEF4 was highly stable on the plasma membrane and inhibited ROP11 diffusion. Our study indicated that ROP-based reaction-diffusion system self-organizes ROP-activated domains, thereby determines the pit pattern of metaxylem vessels.

## Introduction

Pattern formation during plant and animal development is a fundamental issue in biology. The mathematical principle underlying self-organizing pattern formation was first proposed and studied by Turing^1^. This theoretical work showed clearly that the dynamics of simple reactions including at least two chemical species and their diffusion could spontaneously generate periodic spatial distributions. The conditions for pattern formation in this system was initially given as “diffusion-induced instability” (or Turing instability). Later, this mechanism was re-formulated with more intuitive expression “activator-inhibitor system”, which highlighted the importance of local activation by lower diffusion of the activator and long-range inhibition by higher diffusion of the inhibitor^2^. There is increasing evidence that the reaction-diffusion system is involved in various tissue and organ development^3–7^. Several studies combining molecular biology and theoretical modelling have partially revealed the molecular background of reaction-diffusion systems in tissues and organ patterning^8^.

Spatial patterns are also formed in the subcellular space. Rho/Rac GTPases are conserved signalling enzymes that play central roles in directing subcellular patterns^9–13^. They are present in two states: a GTP-bound active form and a GDP-bound inactive form. GTP-bound GTPases interact with their effector proteins to induce downstream signalling, whereas GDP-bound GTPases do not interact with effectors. The switch between GTP/GDP-bound states is controlled by guanine nucleotide exchanging factors (GEFs) and GTP-activating proteins (GAPs). GEFs activate GTPases by exchanging GDP with GTP. Conversely, GAPs promote GTP hydrolysis of the GTPase to produce an inactive GDP-bound form^14^.

An example in which spatial pattern of Rho GTPase directs formation of subcellular patterns is cell wall patterning in plants. We previously demonstrated that pit pattern of secondary cell walls is prefigured by local activation of a Rho/Rac GTPase, ROP11, in differentiating tracheary elements of Arabidopsis metaxylem vessels (hereafter, metaxylem vessel cells). The locally activated ROP11 recruits MIDD1-Kinesin-13A complex, which in turn induces cortical microtubule disassembly, resulting in formation of pits in secondary cell walls^15,16^. Local activation of ROP11 is mediated by a GEF, ROPGEF4, and a GAP, ROPGAP3. Ectopic expression of ROP11, together with ROPGEF4 and ROPGAP3, is sufficient for localized activation of ROP11 and formation of plasma membrane domains^17^. Furthermore, we recently reported that cortical microtubules regulate the shape of ROP-activated plasma membrane domains through IQD13 and CORD1, which confines and releases ROP-activated domains, respectively^18,19^.

As it is still unclear how ROP-activated domains are generated and what determines the pattern of ROP-activated domains, we investigated the mechanism underlying secondary cell wall pit patterning using genetics, reconstructive techniques, and mathematical modelling. *ROPGEF4/7* and *ROPGAP3/4* dose-dependently determined the density of ROP-activated domains, which in turn determined the pattern of the pits formed in the secondary cell walls. Mathematical modelling demonstrated that ROP-activation/inactivation cycle generated ROP domains by reaction-diffusion mechanism. The model predicted that a positive feedback and slow diffusion of ROP-ROPGEF complex were required to generate ROP domains. We found that ROPGEF4 formed a dimer that interacted *in vivo* with activated ROP11, which could provide positive feedback for ROP activation. ROPGEF4 was highly stable on the plasma membrane and inhibited ROP11 diffusion. Our study indicated that ROP-activation/inactivation cycle self-organizes ROP-activated domains *via* a reaction-diffusion mechanism, thereby determines the pit pattern in secondary cell walls.

## Results

### ROPGEFs positively regulate the density and size of secondary cell wall pits

In Arabidopsis metaxylem vessels, *ROPGEF4*, which encodes a plant-specific GEF family protein, is partially required for secondary cell wall pit formation^17^, suggesting that other *ROPGEF* genes also function in metaxylem vessels. We focused on *ROPGEF7*, which is preferentially expressed in xylem vessels^20^. Although there are no T-DNA insertion lines for *ROPGEF7*, we identified an EMS mutant harbouring an AT-insertion between 260 T and 261 G in the second exon of *ROPGEF7*. This mutation caused a frame-shift of the coding sequence, resulting in a premature stop codon in the catalytic domain of ROPGEF7 (Figure S1A). We designated this mutant *ropgef7-1*.

**Figure 1:**
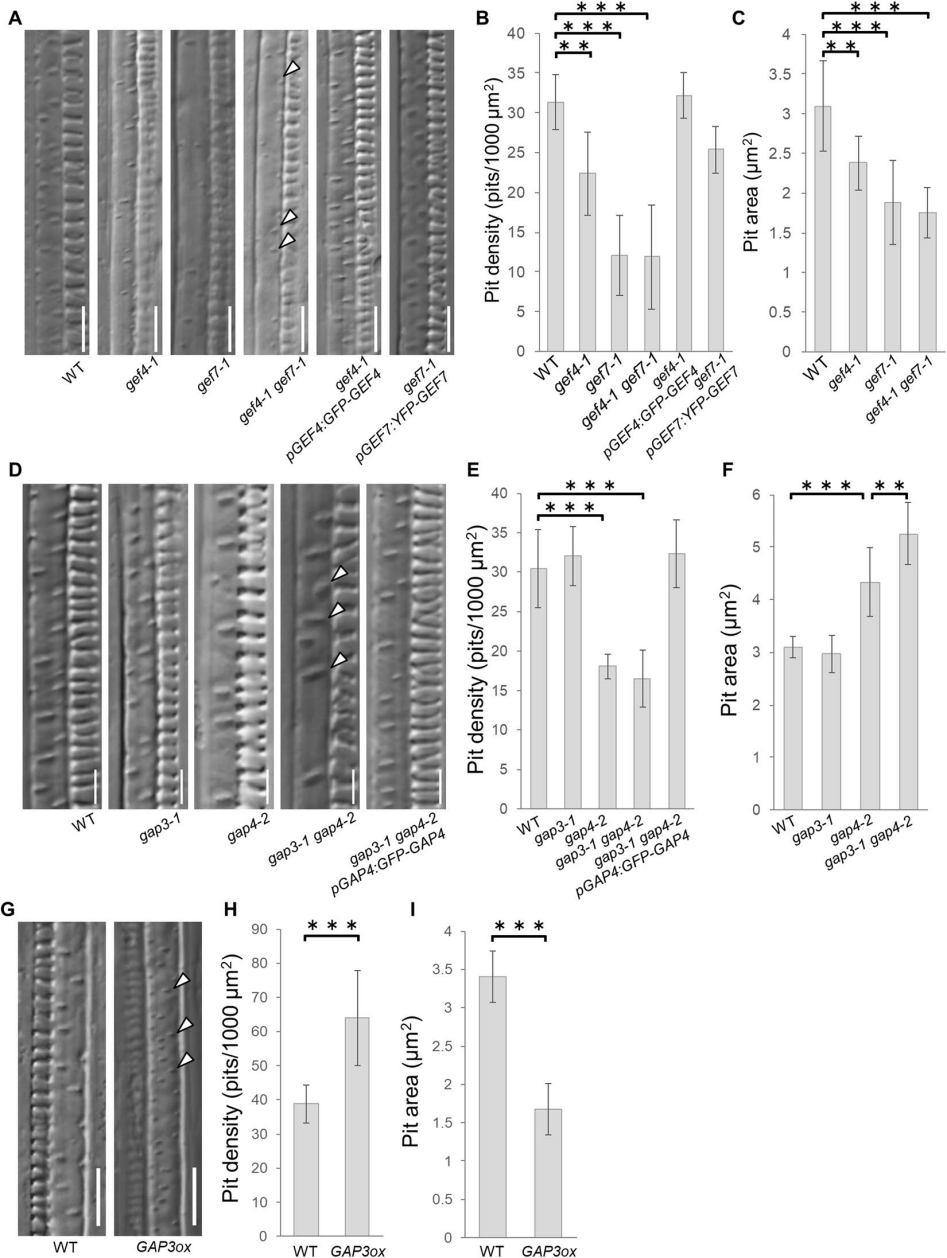
*ROPGEFs* and *ROPGAPs* regulate the secondary cell wall pit patterns. (**A**–**C**) Phenotype of metaxylem vessels in roots of wild-type (WT), *ropgef4-1* (*gef4-1*), *ropgef7-1* (*gef7-1*), and *ropgef4-1 ropgef7-1* plants (**C**), of *ropgef4-1* plants expressing *GFP-ROPGEF4*, and of *ropgef7-1* plants expressing *YFP-ROPGEF7* (**A** and **B**). (**D**–**F**) Phenotype of metaxylem vessels of WT, *ropgap3-1*, *ropgap4-2*, and *ropgap3-1 ropgap4-2* plants (**F**), and *ropgap3-1 ropgap4-2* plants harbouring *pROPGAP4:GFP-ROPGAP4* (**D** and **E**). (**G**–**I**) Phenotype of metaxylem vessels of WT and *ROPGAP3ox* plants. (**A**,**D** and **G**) Differential interference contrast microscopy (DIC) of xylem vessels. Arrowheads indicate secondary cell wall pits. Scale bars = 10 (**A** and **G**) and 5 (**D**) µm. (**B**,**E** and **H**) Density of secondary cell wall pits in roots. Data are means ± SD (n = 10 (**B**) and 12 (**E** and **H**) plants). **P < 0.01; ***P < 0.001; ANOVA with Scheffe’s test (**B** and **E**) and Wilcoxon rank sum test (**H**). (**C**,**F** and **I**) Area of secondary cell wall pits in roots. Data are means ± SD (n = 10 (F) and 12 (**C** and **I**) plants). **P < 0.01; ***P < 0.001; ANOVA with Scheffe’s test (**C** and **F**) and student’s *t* test (**I**).

The secondary cell wall pits of *ropgef4-1*, *ropgef7-1*, and *ropgef4-1 ropgef7-1* mutants were sparser and smaller than those of wild-type plants (Fig. 1A). The density and area of secondary cell wall pits in *ropgef4-1* were about 70% (Fig. 1B) and 80% (Fig. 1C) of those of wild-type plants, respectively. The *ropgef7-1* phenotype resembled *ropgef4-1* but showed a greater reduction in both density and area of secondary cell wall pits (Fig. 1A–C). We also examined the effect of *ROPGEF7* knock-down by introducing *pLexA:ROPGEF7-RNAi* to wild-type plants. The pits in the secondary cell walls of *pLexA:ROPGEF7-RNAi* plants were sparsely distributed and small, indistinguishable from the *ropgef7-1* phenotype (Figures S1E–S1G). We concluded that *ropgef7-1* was a loss-of-function mutant and *ROPGEF7* was required for normal secondary cell wall pit formation. The *ropgef4-1 ropgef7-1* double mutants displayed similar reductions in density and area of secondary cell wall pits to *ropgef7-1* (Fig. 1A–C). The reduced pit phenotypes of *ropgef4-1* and *ropgef7-1* could be rescued by introducing *pROPGEF4:GFP-ROPGEF4* and *pROPGEF7:YFP-ROPGEF7*, respectively. These results demonstrate that both *ROPGEF4* and *ROPGEF7* positively regulated the formation of secondary cell wall pits.

**Figure 2:**
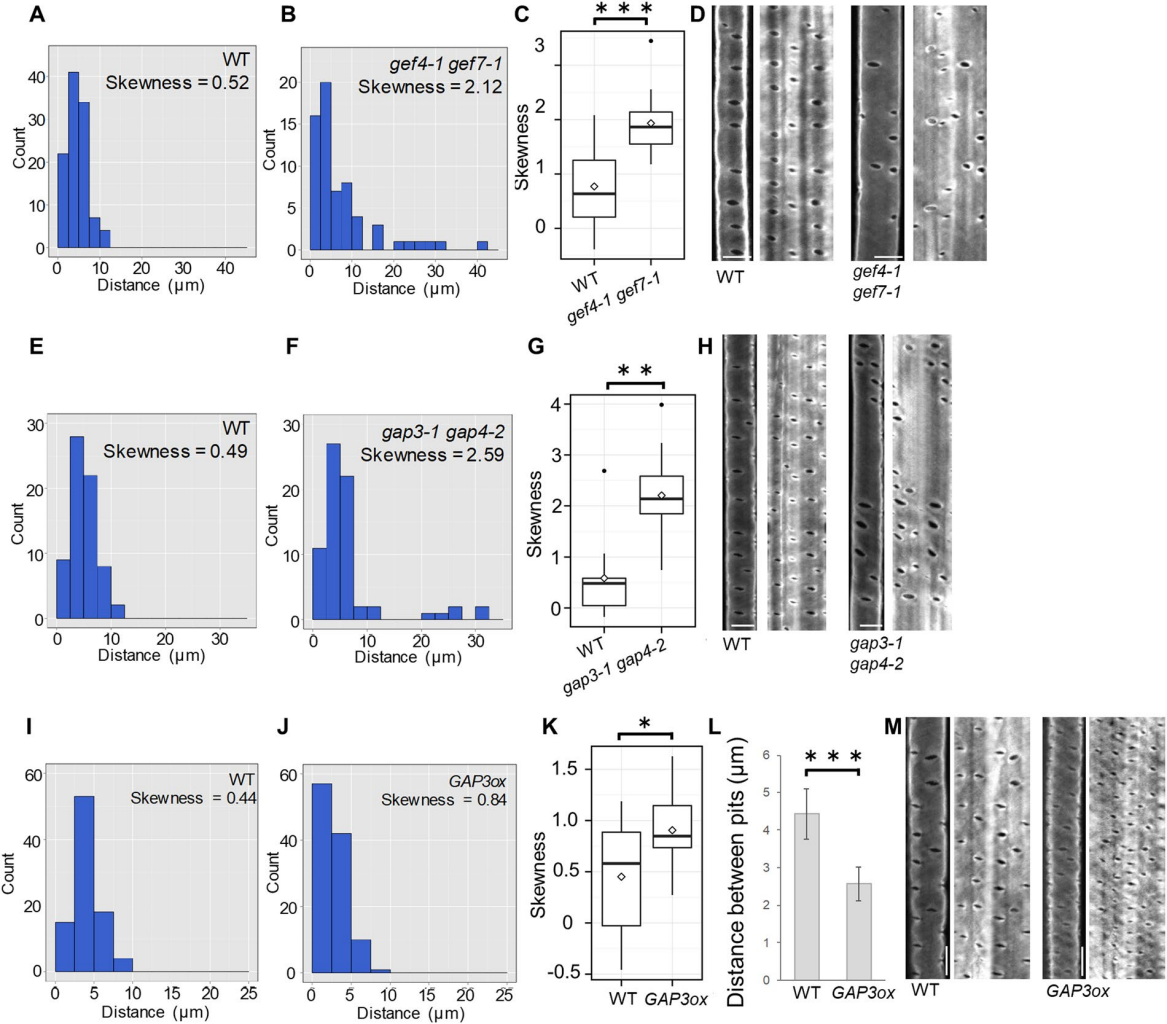
Distributions of secondary cell wall pits in *ropgef* and *ropgap* mutants. Distribution of secondary cell wall pits in WT and *ropgef4-1 ropgef7-1* plants (**A**–**D**), *ropgap3-1 ropgap4-2* plants (**E**–**H**), or *GAP3ox* plants (**I**–**M**). (**A**,**B**,**E**,**F**,**I** and **J**) Histograms showing distances between secondary cell wall pits in WT (**A**,**E** and **I**) and mutant plants (**B**,**F** and **J**). n = 108 (**A**), 64 (**B**), 69 (**E**), 63 (**F**), 90 (**I**), and 110 (**J**) pits. (**C**,**G** and **K**) Degree of skewness of distributions of distances between secondary cell wall pits. White rectangles represent means; black bars represent medians; black dots represent outliers; n = 12 (**C** and **K**) and 10 (**G**) plants; ***P < 0.001, **P < 0.01, *P < 0.05; student’s *t* test (**C** and **K**); Wilcoxon rank sum test (**G**). (**D**,**H** and **M**) Omni-directional images of metaxylem vessel cells. Scale bars = 5 µm. (**L**) Distances between secondary cell wall pits in wild type and *GAP3ox* plants. Data are the mean ± SD (n = 12 plants). ***P < 0.001; student’s *t* test.

In addition to reductions in density and size, pits were distributed less evenly across the secondary cell walls of *ropgef4-1 ropgef7-1* plants than of wild-type plants (Fig. 2A–C). This phenotype was more obvious in the omni-directional image showing 360° of the cell wall area (Fig. 2D). The distance between pits in wild-type plants was less than 10 µm, and usually between 2.5 and 7.5 µm (Fig. 2A); by contrast, there was a broad distribution of distances between the pits in *ropgef4-1 ropgef7-1* mutants that ranged up to 42.5 µm (Fig. 2B), indicating that the pits were less evenly spaced. The distribution of distances between pits in *ropgef4-1 ropgef7-1* double mutants was more highly skewed than in wild-type plants (Fig. 2C). These data suggested that *ROPGEF4* and *ROPGEF7* were required for periodic formation of secondary cell wall pits.

### ROPGAPs positively regulate the pit density, but negatively regulate pit size

ROPGAP3 is localized in the plasma membrane of the secondary cell wall pits, and is required for local activation of ROP11^17^. To investigate the roles of *ROPGAP3* in pitted cell wall patterning, we studied the metaxylem vessels of *ropgap3-1* plants, which have a T-DNA insertion in the first exon of *ROPGAP3* (Figure S1A). *ROPGAP3* mRNA levels in *ropgap3-1* were about 10% of those in wild-type plants (Figure S1B); however, we could not find any differences between wild-type and *ropgap3-1* plants in the density (Fig. 1E) or area (Fig. 1F) of secondary cell wall pits (Fig. 1D), probably due to redundancy between *ROPGAP* genes. We therefore focused on *ROPGAP4*, which is preferentially expressed in xylem vessels^20^, and studied *rogap4-2* plants harbouring a T-DNA insertion at the exon of *ROPGAP4* (Figure S1A). Expression of *ROPGAP4* mRNA in *ropgap4-2* mutants was about 15% that in wild-type plants (Figure S1C), and *ropgap4-2* displayed a reduced density (Fig. 1E) and larger size (Fig. 1F) of pits than wild-type plants. The large pit phenotype of *ropgap4-2* was enhanced in *ropgap3-1 ropgap4-2* double mutants, while the sparse pit phenotype of *ropgap4-2* was not affected in *ropgap3-1 ropgap4-2* double mutants (Fig. 1F). Introduction of *pROPGAP4:GFP-ROPGAP4* rescued the pit density phenotype of *ropgap3-1 ropgap4-2* (Fig. 1E). These data indicated that *ROPGAP3* and *ROPGAP4* negatively regulated pit size and that *ROPGAP4* positively regulated pit formation. *ROPGAP3* may also regulated pit formation positively, but the contribution of *ROPGAP3* was likely minor, thereby masked by the action of *ROPGAP4*.

To clarify the role of *ROPGAP3*, we next investigated the effect on secondary cell wall pit formation of *ROPGAP3* over-expression. We fused the *ROPGAP3* coding sequence with the promoter sequence of *IRX3*, which is strongly expressed in xylem vessels^21^, and introduced this construct (*pIRX3:ROPGAP3)* to wild-type plants (*GAP3ox*). Expression of *ROPGAP3* mRNA was 14-fold higher in *GAP3ox* plants than in wild-type plants (Figure S1D). The secondary cell wall pits were denser and smaller in *GAP3ox* plants than in wild-type plants (Fig. 1G–I). These results suggested that, as with *ROPGAP4*, *ROPGAP3* positively regulated pit formation, but negatively regulated pit size

As in the *ropgef4-1 ropgef7-1* double mutant, pits were less evenly distributed in the secondary cell walls of *ropgap3-1 ropgap4-2* than in wild-type plants (Fig. 2H). There was a wider range of distances between pits in *ropgap3-1 ropgap4-2* double mutants than in wild-type plants (Fig. 2E–G). By contrast, the distances between pits in *GAP3ox* plants were smaller and more evenly distributed than in wild-type plants (Fig. 2I–M). These data imply that *ROPGAP3* and *ROPGAP4* were also required for periodic formation of secondary cell wall pits

### ROPGEF and ROPGAP dose-dependently regulate the density of ROP domains

Our genetic analysis indicated that *ROPGEFs* and *ROPGAPs* regulated formation of the pits in secondary cell walls. Given that ROP-activated domains direct formation of secondary cell wall pits^17^, levels of *ROPGEF* and *ROPGAP* expression may determine the pit pattern through regulating the formation of ROP-activated domains. In the *ropgef* and *ropgap* mutants, ROP-activated domains were specifically associated with the altered pit patterns (Figure S2A), implicating that *ROPGEF* and *ROPGAP* expression levels affected the pattern of ROP-activated domains. To determine whether ROPGEFs and ROPGAPs regulated the pattern of ROP-activated domains, we examined the relationship between ROPGEF and ROPGAP expression and ROP domain patterns by using an ectopic reconstruction of ROP-activated domains; *pLexA:ROP11*, *pLexA:ROPGEF4*^*PRONE*^ (the catalytic domain of ROPGEF4), and *pLexA:ROPGAP3*, were co-introduced into tobacco (*Nicotiana benthamiana*) leaves by the syringe-infiltration with *R. radiobacter*. Two days after infiltration, the leaf samples were harvested and inoculated with 2 µM estradiol to simultaneously express the introduced genes. This enables the formation of numerous plasma membrane domains marked with ROPGEF4^PRONE^ and activated ROP11 in the tobacco leaf epidermal cells^17^. As full length ROPGEFs auto-inhibit their GEF activity^22^, ROPGEF4^PRONE^ was used instead of full length ROPGEF4. ROPGEF4^PRONE^ rescued the pit pattern phenotype of *ropgef4-1* to the same extent as full length ROPGEF4 did (Figs 1B, S2B and S2C) and localized to secondary cell wall pits in roots (Figure S2D). ROPGEF4^PRONE^ was therefore fully functional in pit patterning. ROP11 was locally activated just around ROPGEF4^PRONE^ domains (Figure S2E)^17^, thus patterns of ROPGEF4 ^PRONE^ domains indicated those of ROP-activated domains.

**Figure 3:**
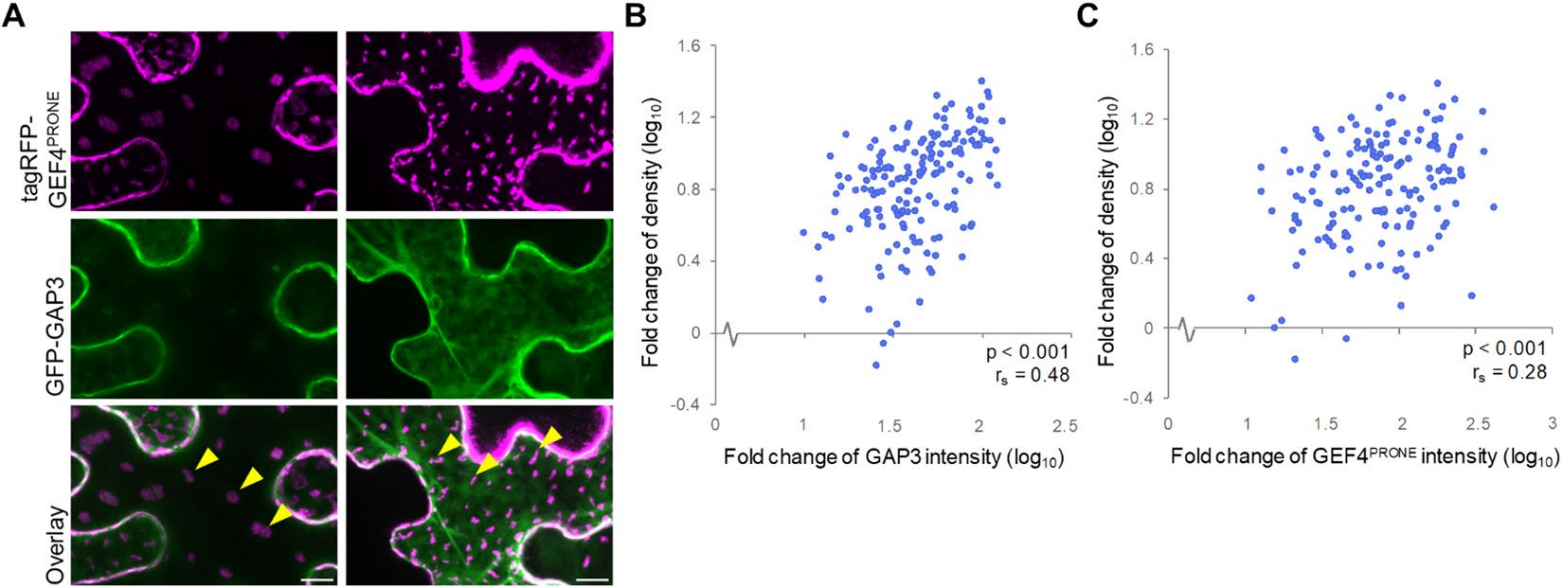
Effects of ROPGAP3 and ROPGEF4 levels on ROP domain patterning. (**A**) Formation of sparse (left) and dense (right) ROP domains (yellow arrowheads) in *N. benthamiana* leaf epidermal cells. *pLexA:tagRFP-ROPGEF4*^*PRONE*^ and *pLexA:GFP-ROPGAP3* were introduced together with *pLexA:ROP11*. Scale bars = 10 µm. (**B** and **C**) Plots of (**B**) GFP-ROPGAP3 intensity and ROP domain density; (**C**) tagRFP-ROPGEF4^PRONE^ intensity and ROP domain density. n = 165 cells. r_s_: Spearman’s rank correlation coefficient.

Following induction of *ROP11*, *tagRFP-ROPGEF4*^*PRONE*^, and *GFP-ROPGAP3*, numerous ROPGEF4 domains formed on the plasma membrane (Fig. 3A). We recorded images from over 100 cells expressing various levels of tagRFP-ROPGEF4^PRONE^ and GFP-ROPGAP3, and quantified the mean intensity of GFP-ROPGAP3 and tagRFP-ROPGEF4^PRONE^, as well as the density of ROPGEF4^PRONE^ domains in each cell. There was a positive correlation between domain density and mean intensity of GFP-ROPGAP3 (Fig. 3B) and tagRFP-ROPGEF4^PRONE^ (Fig. 3C). These correlations suggested that the density of ROPGEF4^PRONE^ domains was coupled with the expression levels of ROPGEF4^PRONE^ and ROPGAP3. This result was consistent with the pit pattern phenotypes of the *ropgef4* and *ropgef7* mutants, *ropgap3 ropgap4* double mutants, and *ROPGAP3*-over-expressing plants. These data suggested that ROPGAP3 and ROPGEF4 determined the pit pattern of secondary cell walls by regulating the pattern of ROP-activated domains.

We next tested whether ROPGEF7^PRONE^ mediates formation of ROP-activated domains. Previously we reported that ectopic expression of ROPGEF7^PRONE^ together with ROP11 and ROPGAP3 did not generate domains in leaf epidermal cells^17^. As ROPGEFs have different affinity to different ROPs, we assumed that ROPGEF7 activates ROPs other than ROP11. We therefore re-examined the ability of ROPGEF7^PRONE^ to form domains by using ROP2, which is another ROP expressed in xylem vessels^20^. ROPGEF7^PRONE^ formed domains on the plasma membrane when expressed with ROPGAP3 and ROP2 (Figure S2F). This suggested that ROPGEF7 also regulate patterns of ROP-activated domains to direct formation of secondary cell wall pits.

### Mathematical Model and Analysis

Although our genetic analysis suggested that ROPGEF and ROPGAP determine the pit pattern through regulating the pattern of ROP domains, it is unclear how the ROP domains are formed. Considering that the density of ROP domains varied with the levels of ROPGEF and ROPGAP expression, it is plausible that ROP domains were self-organized through the action of ROPGEFs and ROPGAPs.

**Figure 4:**
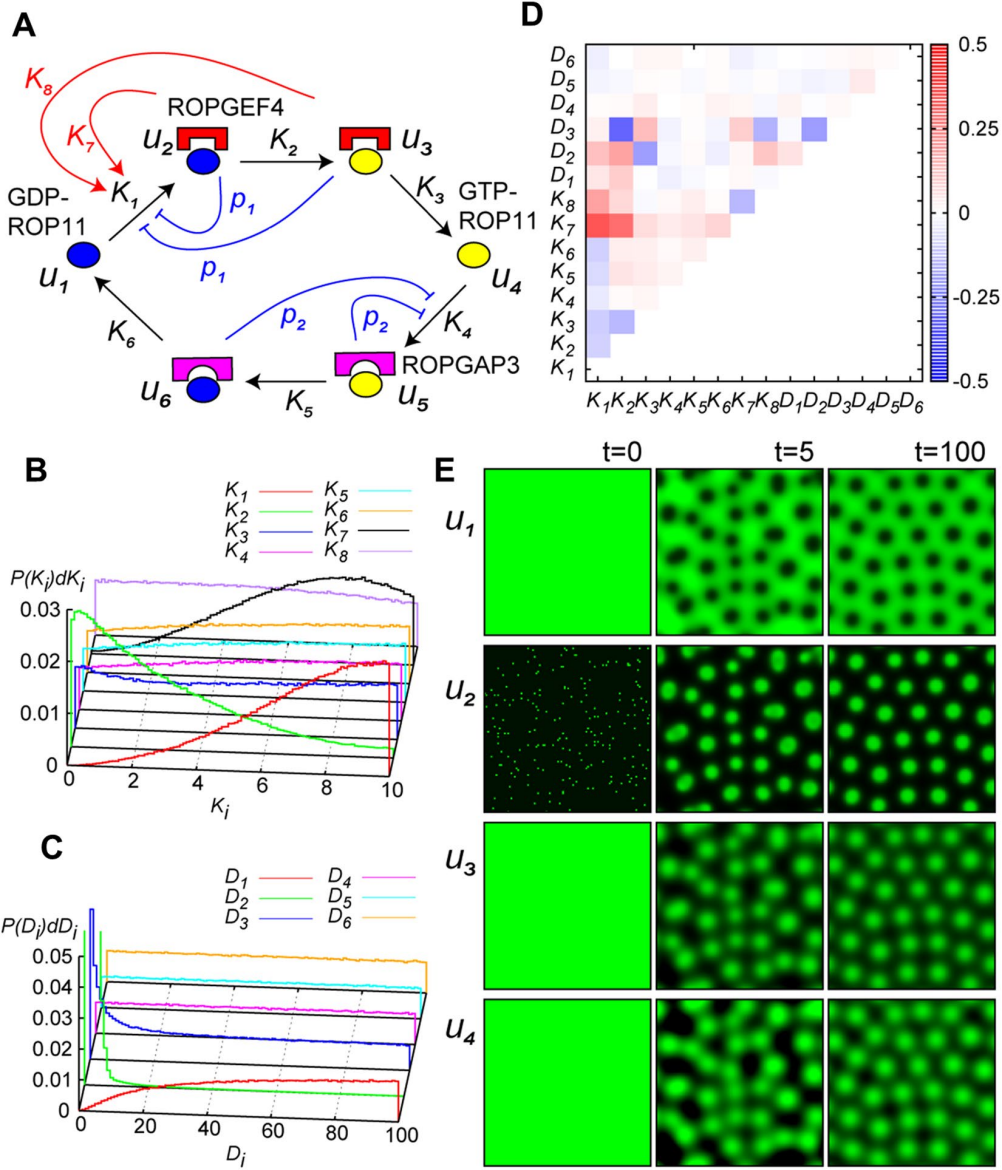
Mathematical analysis of pattern formation of ROP activity. (**A**) Schematic representation of reaction network for dynamics of concentration of ROP states. *u*_*i*_(***x***, *t*)s are *u*_1_: GDP-ROP; *u*_2_: GDP-ROP ROPGEF complex; *u*_3_: GTP-ROP ROPGEF complex; *u*_4_: GTP-ROP; *u*_5_: GTP-ROP ROPGAP complex; and *u*_6_: GDP-ROP ROPGAP complex. *k*_*i*_ are parameters associated to reaction rates, ${k}_{i}=\partial {r}_{i}/\partial {u}_{i} > 0$ (*i* = 1, …, 6). Parameters of regulation are given as *k*_7_ = ∂*r*_1_/∂*u*_2_, *k*_8_ = ∂*r*_1_/∂*u*_3_, ${p}_{1}=\partial {r}_{1}/\partial {F}_{G}\cdot \partial {F}_{G}/\partial {u}_{i}$ (*i* = 2, 3), and ${p}_{2}=\partial {r}_{4}/\partial {F}_{P}\cdot \partial {F}_{P}/\partial {u}_{i}$ (*i* = 5, 6). (**B** and **C**) Distribution of successful parameter sets: (**B**) Distribution of ${k}_{1} \sim {k}_{8}$; and (**C**) ${D}_{1} \sim {D}_{6}$. (**D**) Pearson’s product-moment correlation among parameter values in successful sets. (**E**) Numerical simulations of dynamics of pattern formation of ROP activities represented by *u*_1_, *u*_2_, *u*_3_, and *u*_4_.

Reaction-diffusion system^1^ is a potential mechanism capable of self-organization of the ROP domains. To examine this hypothesis, we developed a mathematical model of pattern formation of ROP activities on the plasma membrane. In this model, we considered all the reactions involving ROP, binding of GAP or GEF molecules with ROPs, GDP/GTP exchange (GEF activity), and GTP hydrolysis (GAP activity), which constituted a circular state-transition network, illustrated in Fig. 3A (black arrows). The mobility of ROP molecules on the plasma membrane was modelled as diffusion in a two-dimensional space, and we have the following reaction-diffusion (RD) model for the dynamics of ROP (See also Fig. 4A):

$$\begin{array}{rcl}\frac{\partial {u}_{1}}{\partial t} & = & {D}_{1}{{\nabla }}^{2}{u}_{1}+{r}_{6}({u}_{6})-{r}_{1}({u}_{1},{u}_{2},{u}_{3},{F}_{G})\\ \frac{\partial {u}_{2}}{\partial t} & = & {D}_{2}{{\nabla }}^{2}{u}_{2}+{r}_{1}({u}_{1},{u}_{2},{u}_{3},{F}_{G})-{r}_{2}({u}_{2})\\ \frac{\partial {u}_{3}}{\partial t} & = & {D}_{3}{{\nabla }}^{2}{u}_{3}+{r}_{2}({u}_{2})-{r}_{3}({u}_{3})\\ \frac{\partial {u}_{4}}{\partial t} & = & {D}_{4}{{\nabla }}^{2}{u}_{4}+{r}_{3}({u}_{3})-{r}_{4}({u}_{4},{F}_{P})\\ \frac{\partial {u}_{5}}{\partial t} & = & {D}_{5}{{\nabla }}^{2}{u}_{5}+{r}_{4}({u}_{4},{F}_{P})-{r}_{5}({u}_{5})\\ \frac{\partial {u}_{6}}{\partial t} & = & {D}_{6}{{\nabla }}^{2}{u}_{6}+{r}_{5}({u}_{5})-{r}_{6}({u}_{6}).\end{array}$$

In the RD equations, *u*_*i*_(***x***, *t*)s are time-variable spatial distribution of concentrations of *u*_1_: GDP-ROP; *u*_2_: GDP-ROP ROPGEF complex; *u*_3_: GTP-ROP ROPGEF complex; *u*_4_: GTP-ROP; *u*_5_: GTP-ROP ROPGAP complex; and *u*_6_: GDP-ROP ROPGAP complex. *r*_*i*_ is reaction rate with *K*_*i*_ = ∂*r*_*i*_/∂*u*_*i*_ > 0 (*i* = 1, …, 6); *F*_*G*_ and *F*_*P*_ are concentrations of free cytoplasmic GEF and GAP molecules, respectively. At a glance, the network structure of the RD model differs from that of typical system showing Turing instability, like activator-inhibitor system. In the following we examined the condition of the network structure for generating periodic spatial patterns.

As feedback regulation is considered to be important for pattern formation in RD systems^23^, we examined the effects of two mechanisms that may act as feedback in the ROP-activation/inactivation cycle. Firstly, due to conservation of the total amount of GEF, an increase in GEF-bound states in a cell will reduce the number of free GEF molecules in the cytoplasm, which will in turn reduce the GEF-binding reaction. This negative regulation of GEF-binding reaction by free GEF was incorporated into the model by setting ${p}_{1}\equiv \partial {r}_{1}/\partial {F}_{G}\cdot \partial {F}_{G}/\partial {u}_{i}\le 0$ (*i* = 2, 3). Similarly, negative regulation of the GAP-binding reaction by free GAP was modelled as ${p}_{2}\equiv \partial {r}_{4}/\partial {F}_{P}\cdot \partial {F}_{P}/\partial {u}_{i}\le 0$ (*i* = 5, 6) (blue curves in Fig. 4A). Secondly, we considered GEF dimerization. ROPGEFs form homo-dimer *in vitro*, in which each subunits interact and catalyse ROP molecules^24^. Once a subunit of ROPGEF dimer interacts with ROP, the other subunit of the dimer will activate another ROP nearby. This will enhance the reaction rate of ROP-GEF binding by producing a locally higher concentration of ROP-GEF complexes. This positive feedback from GEF-bound states to GEF-binding reaction was modelled by setting ${K}_{7}\equiv \partial {r}_{1}/\partial {u}_{2}\ge 0$, ${K}_{8}\equiv \partial {r}_{1}/\partial {u}_{3}\ge 0$ (red curves in Fig. 4A). Based on these arguments we developed three models: (A) Closed-circuit model without regulations (*p*_1_ = 0, *p*_2_ = 0; *K*_7_ = *K*_8_ = 0); (B) Inhibition from conserved quantities (${p}_{1} < 0$, ${p}_{2} < 0$; *K*_7_ = *K*_8_ = 0); and (C) Positive feedback from dimerization (*p*_1_ = 0, *p*_2_ = 0; ${K}_{7} > 0$, ${K}_{8} > 0$). We compared dynamical properties originated from difference in network structures.

We analysed the models using three methods: (i) mathematical analysis; (ii) numerical analysis; and (iii) numerical simulation. In the mathematical analysis, we adopt “structural analysis” where the structural conditions of the reaction network for pattern formation was determined based on Turing instability (see Methods). We obtained the following strong result: the closed-circuit model (A) and the inhibition model (B) never show Turing instability from their structures, while the positive feedback model (C) can represent the Turing instability. This result implies that the negative regulation by the conserved quantities, GEF and GAP, did not contribute to pattern formation. The presence (${p}_{1} < 0$ or ${p}_{2} < 0$) or absence (*p*_1_ = 0 or *p*_2_ = 0) of this regulation did not influence Turing instability. On the other hand, positive feedback from GEF-bound states (*u*_2_ and *u*_3_) on the GEF-binding reaction (*r*_1_) was necessary for pattern formation (Table S1). If we removed the positive feedback regulation (*K*_7_ = *K*_8_ = 0) from the model, the system never satisfied the condition of Turing instability, although the condition could be met in the original model with such regulation (${K}_{7},{K}_{8} > 0$).

Using the network including positive feedback, we performed exhaustive searches to examine the quantitative conditions of the parameter values for pattern formation. Parameter values were changed randomly, and the condition of Turing instability was examined numerically for each parameter set. By examining the statistical properties of successful parameter sets, we identified a quantitative condition of parameters. Figure 4B shows that the coefficient of reaction 1 was high, that of reaction 2 was low in the successful parameter sets, indicating the importance of the rates of ROP-GEF binding and GEF reaction. Figure 4C shows that the diffusion coefficient of *u*_2_ (and *u*_3_) were small, indicating that the diffusion rate of ROP-GEF complex must be slow to form ROP-activated domains.

This tendency can be understood by analogy with the activator-inhibitor system. The Turing instability condition requires a smaller diffusion of activator than other factors; thus *u*_2_ (or *u*_3_) with positive feedback (${K}_{7},{K}_{8} > 0$) plays the role of an activator in the reaction network while *u*_4_ − *u*_6_ are inhibitors. The correlations between parameters (Fig. 4D) enabled additional interpretations of the properties of the system. The negative correlation between *K*_2_ and *D*_3_ suggested that increasing *K*_2_ resulted in a lower total amount of *u*_2_, and required *u*_3_, rather than *u*_2_, to take the role of activator with smaller *D*_3_; the negative correlation between *K*_3_ and *D*_2_ could be interpreted inversely. These correlations implied that either *u*_2_ or *u*_3_ had to be the activator in this system.

Finally, we calculated the dynamical change in spatial patterns of active ROP using numerical simulation of the model including all feedback regulations (${p}_{1} < 0$, ${p}_{2} < 0$; ${K}_{7} > 0$, ${K}_{8} > 0$). Figure 4E and Movie S1 show an example of the periodic domains of activated ROPs obtained using one of the successful parameter sets. The obtained patterns resembled the observed distributions of active ROP in experiments (Figure S2E)^17^.

### *In vivo* interaction between ROP11 and ROPGEFs/ROPGAPs

Our mathematical analysis demonstrated that the ROP-activation/inactivation cycle could generate ROP-activated domains by the reaction-diffusion mechanism. The model predicted positive feedback from GEFs and slower diffusion of GEF-ROP complex. To determine whether ROPGEF4 was involved in the feedback reaction, we tested the *in vivo* interactions of ROPGEF4^PRONE^ with ROP11, ROP11^G17V^ (a constitutive GTP-bound active form), ROP11^T22N^ (a constitutive GDP-bound inactive form of ROP11), and ROPGEF4^PRONE^ using a bimolecular fluorescence complementation (BiFC) assay and fluorescent resonance energy transfer (FRET) in tobacco leaf epidermal cells.

**Figure 5:**
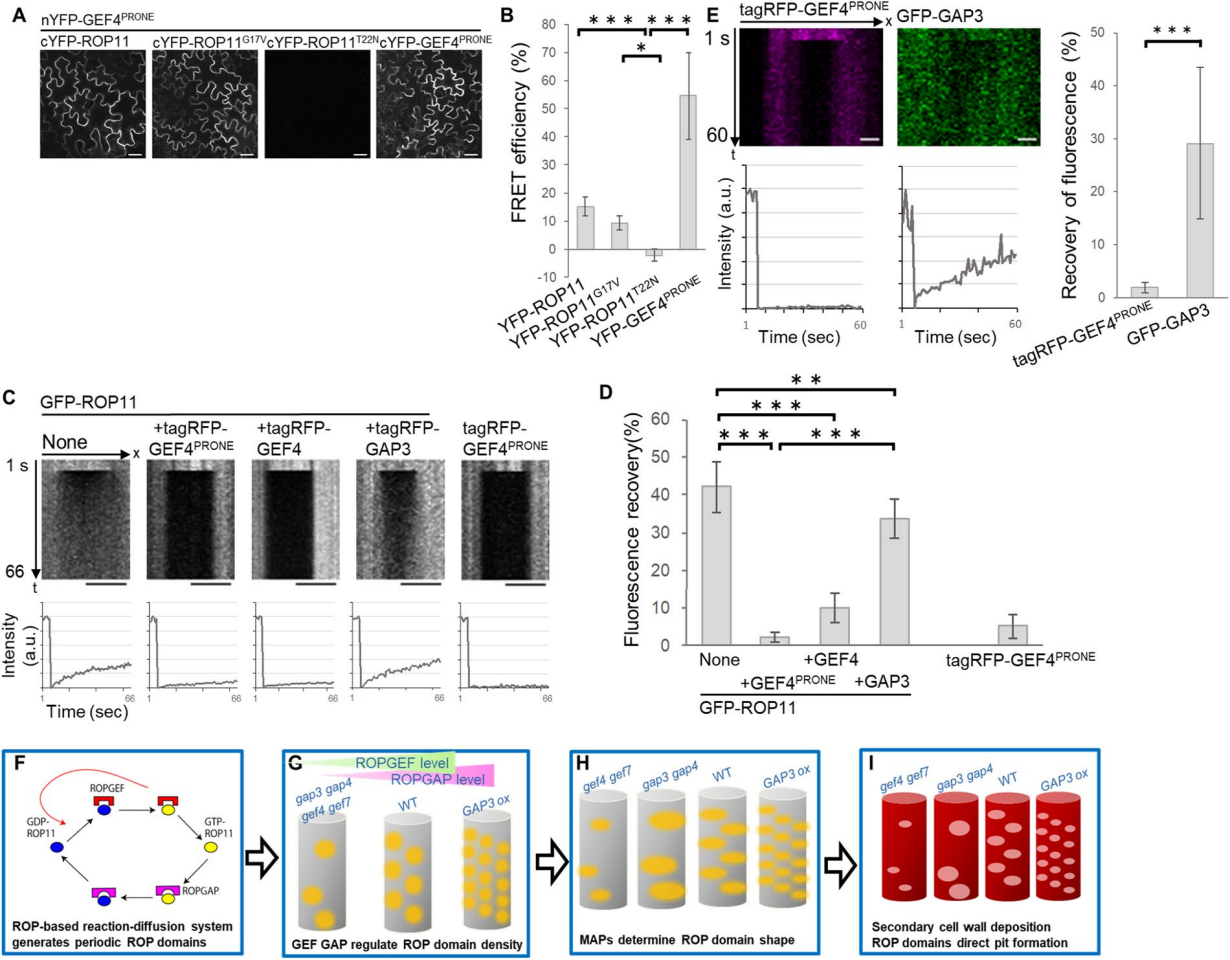
ROPGEF4^PRONE^ interacts with GTP-ROP11 and inhibits the diffusion of ROP11 in tobacco leaf epidermal cells. (**A**) BiFC assay between nYFP-ROPGEF4^PRONE^ and cYFP-ROP11, cYFP-ROP11^G17V^, cYFP-ROP11^T22N^, or cYFP-ROPGEF4^PRONE^. Scale bars = 20 µm. (**B**) FRET efficiency between CFP-ROPGEF4^PRONE^ and YFP-ROP11 derivatives or YFP-ROPGEF4^PRONE^. Data are means ± SD (n = 12). *P < 0.05; ***P < 0.001; ANOVA with Scheffe’s test. (**C** and **D**) FRAP analysis of GFP-ROP11 and tagRFP-ROPGEF4^PRONE^. GFP-ROP11 was expressed alone (NONE) or together with tagRFP-ROPGEF4^PRONE^, tagRFP-ROPGEF4, or tagRFP-ROPGAP3 in tobacco leaf epidermal cells. Kymograph of photo-bleached area at the mid plane of leaf epidermal cells (C; top); intensity plots of photo-bleached area (C; bottom); and fluorescence recovery percentage 60 s after photo-bleaching (**D**) are shown. In (**C**), t indicates time after the onset of scan. x indicates position along the plasma membrane. Photo-bleach was executed at 6 s. Data are means ± SD (n = 12). Bar  = 5 µm. ***P < 0.001; **P < 0.01; ANOVA with Scheffe’s test. Note that the recovery of GFP signals at the edges of photo-breached area is faster than that at the centre of the area in C (None and +tagRFP-GAP3). (**E**) FRAP analysis of tagRFP-ROPGEF4^PRONE^ and GFP-ROPGAP3 in ROP11-activated domains. ROP11 was co-expressed with tagRFP-ROPGEF4^PRONE^ and GFP-ROPGAP3. Kymograph of photo-bleached area (left top) and intensity of photo-bleached area (left bottom), and recovery of fluorescence at 60 s after photo-bleaching (right). Data are means ± SD (n = 15). Scale bar = 1 µm. ***P < 0.001; Welch’s *t* test. (**F** to **I**) Schematic model of secondary cell wall pit patterning. (**F**) The ROP-activation cycle generates periodic ROP-activated domains by reaction-diffusion mechanism. Positive feedback is provided by ROPGEF4 dimers interacting with GTP-ROP11. (**G**) ROPGEFs and ROPGAPs determine the density of ROP-activated domains. (**H**) The size and shape of ROP-activated domains are determined through the action of microtubule-associated proteins (MAPs). ROP-activated domains promote depletion of cortical microtubules. (**I**) Secondary cell walls are deposited on the area outside of the ROP11-activated domains, resulting in the formation of secondary cell wall pits.

We first performed the BiFC assay. YFP signals were detected when nYFP (N-terminal half of YFP)-ROPGEF4^PRONE^ was expressed together with cYFP (C-terminal half of YFP)-ROP11, cYFP-ROP11^G17V^, or cYFP-ROPGEF4^PRONE^, but YFP signals were not detected when nYFP-ROPGEF4^PRONE^ was co-expressed with cYFP-ROP11^T22N^ (Fig. 5A), suggesting that ROPGEF4 ^PRONE^ preferentially interacted with GTP-ROP11 and with ROPGEF4^PRONE^ itself.

We next carried out FRET analysis that could detect direct interaction between proteins *in vivo*. The mean FRET efficiency between CFP-ROPGEF4^PRONE^ and YFP-ROP11 (15%), YFP-ROP11^G17V^ (9%), or YFP-ROPGEF4^PRONE^ (55%) was significantly higher than that between CFP-ROPGEF4^PRONE^ and YFP-ROP11^T22N^ (−2%) (Fig. 5B). Our data suggested that ROPGEF4^PRONE^ forms dimer and directly bind to GTP-ROP11, besides the regular binding for GDP-GTP exchange. In this context, activated GTP-ROP11 could recruit a ROPGEF4^PRONE^ dimer, which in turn activates another, nearby ROP11. This could result in a positive feedback of ROP activation.

### ROPGEF4^PRONE^ is highly stable on the plasma membrane

To investigate diffusion of ROP11 and ROPGEF4^PRONE^ at the plasma membrane, we analysed fluorescence recovery after photo-bleaching (FRAP) of ROP11 and ROPGEF4^PRONE^. We introduced *pLexA:GFP-ROP11* or *pLexA:tagRFP-ROPGEF4*^*PRONE*^ into tobacco leaf epidermal cells, photo-bleached regions of the plasma membrane at the mid plane of the cells, and recorded the recovery of the fluorescence for 60 s (Fig. 5C). GFP-ROP11 fluorescence recovered to 40% of pre-bleaching intensity by 60 s after bleach (Fig. 5D). The dynamic recovery or GFP-ROP11 fluorescence could be due to lateral diffusion and/or cytoplasmic shuttling of ROP11. The faster recovery at the edges of photo-bleached region than at the central region (Fig. 5C) suggested that a significant amount of ROP11 molecules diffused laterally on the plasma membrane. By contrast, there was little recovery of tagRFP-ROPGEE4^PRONE^ fluorescence, suggesting that ROPGEF4^PRONE^ was stable on the plasma membrane.

As ROPGEF4^PRONE^ interacted with ROP11 *in vivo*, we hypothesized that ROPGEF4^PRONE^ influenced the dynamics of ROP11. We co-introduced *GFP-ROP11* and *tagRFP-ROPGEF4*^*PRONE*^ into tobacco leaf epidermal cells and analysed the recovery of GFP-ROP11 signals after photo-bleaching. Recovery of GFP-ROP11 was dramatically inhibited in the presence of ROPGEF4^PRONE^ (Fig. 5C and D). As with ROPGEF4^PRONE^, tagRFP-ROPGEF4 (the full length version of ROPGEF4) also inhibited recovery of GFP-ROP11 fluorescence (Fig. 5C and D), suggesting that ROPGEF4 inhibited diffusion of ROP11. In contrast to ROPGEF4, tagRFP-ROPGAP3 slightly inhibited the recovery of GFP-ROP11, suggesting that ROPGAP3 also affected the diffusion of ROP11 but much more weakly than ROPGEF4. Consistent with this, ROPGAP3 was entirely cytoplasmic, thus the diffusion of ROPGAP3 could not be studied using FRAP analysis.

We next quantified the recovery of tagRFP-ROPGEF4^PRONE^ in the ROP-activated domains. *TagRFP-ROPGEF4*^*PRONE*^ was introduced, together with *ROP11* and *GFP-ROPGAP3*, into tobacco leaf epidermal cells. GFP-ROPGAP3 was faintly localized to the plasma membrane; thus both tagRFP-ROPGEF4^PRONE^ and GFP-ROPGAP3 were subjected to FRAP analysis. TagRFP-ROPGEF4^PRONE^ did not recover but GFP-ROPGAP3 recovered to 30% of pre-bleaching intensity (Fig. 5E). These data suggested that ROPGEF4^PRONE^ formed a stable domain in the plasma membrane, while ROPGAP3 was dynamic.

### ROPGAP3 interacts with the active form of ROP11

Our model predicted feedback from ROPGEF4^PRONE^, and we subsequently showed that ROPGEF4^PRONE^ could produce feedback for ROP activation. We next determined whether ROPGAPs were also involved in a feedback reaction. As ROPGAP promotes production of GDP-ROP, ROPGAP had to interact with GDP-ROP to provide positive feedback. We conducted a BiFC assay to test interactions between ROPGAP3 and ROP11 in tobacco leaf epidermis. YFP signals were detected when cYFP-ROPGAP3 was expressed with nYFP-ROP11^G17V^, but not when cYFP-ROPGAP3 was expressed with nYFP-ROP11 or nYFP-ROP11^T22N^ (Figure S3A). The mean FRET efficiency between CFP-ROPGAP3 and YFP-ROP11 (18%) or YFP-ROP11^G17V^ (39%) was significantly higher than that between CFP-ROPGAP3 and YFP-ROP11^T22N^ (−0.2%) (Figure S3B). These data suggested that ROPGAP3 specifically interacted with GTP-ROP11 *in vivo*, a result consistent with previous reports that ROPGAPs interact through their Cdc42/Rac interactive binding motif (CRIB, an effector-binding domain) and GAP domain with the active form of ROPs to inactivate them^25,26^. We therefore concluded that ROPGAP3 was not part of the feedback reaction and acted simply as a GAP *in vivo*.

## Discussion

In metaxylem vessel cells, domains of activated ROPs direct secondary cell wall pit formation^17^. Ectopic expression of ROP11 together with ROPGEF4 and ROPGAP3 induced formation of ROP-activated domains; this suggested that ROP11-activation/inactivation cycle could spontaneously generate ROP-activated domains^17^. Our mathematical model using reaction-diffusion equations showed that the ROP-activation/inactivation cycle can generate the ROP11-activated domains. Domain generation required feedback from GEF and slow diffusion of the GEF-ROP complex. We showed that ROP11 form a dimer and interacts directly with GTP-ROP11 *in vivo*. This could provide positive feedback to ROP activation, as the dimer would be recruited to GTP-ROP11 and subsequently activate another ROP11 nearby. We also showed that ROPGEF4^PRONE^ was stable on the plasma membrane and inhibited diffusion of ROP11. Thus our results supported a model in which the ROP-activation/inactivation cycle generated ROP domains by the reaction-diffusion mechanism (Fig. 5F).

Although our observation indicated that ROPGEF4 provides positive feedback by direct binding of ROPGEF4 dimer to GTP-ROP11, it would be also possible that scaffold proteins mediate positive feedback of ROPGEF4. Several receptor-like kinases (RLKs) interact with ROPGEFs, including ROPGEF4^27–30^. Cell walls affect the diffusion rate of membrane proteins, including RLKs^31^. ROPGEF4^PRONE^ was stable at the plasma membrane. ROPGEF4 may interact with RLKs to form the slow-diffusive complex. In pollen tubes, a RLK, PRK2, phosphorylates ROPGEF1, which in turn activates ROP1^32^. The activated GTP-ROP1 interacts with both ROPGEF1 and PRK2, which promotes ROPGEF-PRK2 interaction, resulting in a positive feedback for ROP activation^32,33^. Similarly, RLKs may also act as scaffolds to provide positive feedback from ROPGEF4 to ROP11 activation.

In contrast to ROPGEF4, ROPGAPs are not likely to provide positive feedback to ROP inactivation. Our mathematical model generated ROP-activated domains without setting positive feedback to ROP inactivation. In the model, ROPGAPs globally inactivated the diffusive ROP at the plasma membrane. In pollen tubes, ROPGAPs maintain the ROP-activated domain through global inactivation of diffusive ROP propagating from the apex to lateral region^34^. Similarly, in metaxylem vessel cells, ROPGAPs may maintain the ROP-activated domains through global inactivation of diffusive ROP propagating outward from the ROPGEF domains.

ROP-based reaction-diffusion system can explain not only formation of ROP11 domains but also pattern formation of the domains. We showed that secondary cell wall pits were formed in periodic patterns and that loss of *ROPGEF*s or *ROPGAPs* impaired the periodic pattern of the pits. Our mathematical model showed that ROP-reaction generated ROP-activated domains in a periodic pattern in the absence of prepattern, consistent with the nature of reaction-diffusion system. Thus, it is plausible that, in differentiating metaxylem vessel cells, ROP-activation/inactivation cycle self-organizes ROP-activated domains in a periodic pattern, then the domains function as the first positional cue to direct formation of periodic pits in secondary cell walls (Fig. 5F).

Our genetic analysis showed that *ROPGEFs* and *ROPGAPs* positively regulate the density of secondary cell wall pits. We also showed that the density of ROP-activated domains is determined by levels of ROPGEFs and ROPGAPs, suggesting that ROPGEFs and ROPGAPs determines the pattern of pits by regulating the pattern of ROP-activated domains (Fig. 5G). Given that ROP-activation/inactivation cycle self-organizes ROP domains by reaction-diffusion mechanism, it is plausible that the density of the domain is also determined through reaction-diffusion mechanism. In reaction-diffusion system, domain patterns of different density can be formed by varying the diffusion rate of the components^35^. The inhibitory effects on ROP diffusion rate of ROPGEFs and ROPGAPs may contribute to the density of ROP domains. Precise modelling of the behaviour of ROPGEFs and ROPGAPs should reveal the mechanism by which ROPGEF and ROPGAP levels determine the density of pits.

Our observation suggested that *ROPGEFs* and *ROPGAPs* positively and negatively regulate the pit size, respectively. We previously showed that the pit size is positively regulated by the microtubule-depolymerizer, Kinesin-13A. Kinesin-13A is recruited to the ROP-activated domains via MIDD1^15^. Thus it is plausible that *ROPGEFs* and *ROPGAPs* indirectly determine the pit size by regulating the amount of active ROP11 to recruit Kinesin-13A. This notion is consistent with the pit size phenotype of *ropgef* and *ropgap* mutants, which could decrease and increase the amount of active ROP11, respectively. In addition to Kinesin-13A, IQD13 and CORD1 regulate the size and shape of ROP domains through cortical microtubules surrounding the ROP domains, which in turn determine the size and shape of secondary cell wall pits^18,19^ (Fig. 5H). Once the pattern and shape of ROP domains are determined, secondary cell walls would be deposited along the cortical microtubules lying at the outside of the ROP domains, resulting in the formation of secondary cell wall pits (Fig. 5I). Thus, the ROP-activation/inactivation reaction governs the formation of the pitted pattern of secondary cell walls.

In yeasts, Cdc42 GTPase directs polarity formation through reaction-diffusion mechanism^36^. Cdc42 GTPase is activated by a GEF, Cdc24, and then the activated Cdc42 recruits Cdc24 through the PAK-Bem1 scaffold, providing positive feedback^37^. In this system, the reaction components diffuse rapidly in the cytoplasm of tiny intracellular space, which causes competition between domains, resulting in formation of single domain for budding within the cell^38^. In contrast to yeast model, in metaxylem vessel cells, ROP-activation cycle generated co-existing multiple domains depending on the levels of ROPGEFs and ROPGAPs to direct pitted secondary cell wall pattern. Our study revealed that ROP/Rho GTPase-reaction cycle could not only generate polarity but self-organize periodic patterns of plasma membrane domains. The ROP/Rho-driven reaction-diffusion system may thus contribute to a broad range of subcellular pattern formation.

## Methods

### Plant materials and growth conditions

All Arabidopsis plants used in this study were in the Col-0 background. The T-DNA insertion lines, *ropgap3-1* (SALK_056521) and *ropgap4-2* (SALK_152535), were obtained from the Arabidopsis Biological Resource Center (ABRC). *ropgef4-1* (SAIL_184_c08) has been reported previously^17^; *ropgef7-1* was generated by EMS mutagenesis in this study.

Seedlings were grown on 0.5× MS agar medium containing 0.2% sucrose at 22 °C under constant light. For the observation of root metaxylem vessels, 7-day-old seedlings were used. DIC images of xylem vessels were acquired at the area that are 8 to 12 mm away from the tip of primary roots.

### Quantitative RT-PCR

Total RNA was purified from the roots of Arabidopsis using the Favor Prep Plant total RNA purification Mini Kit (FAVORGEN, http://www.favorgen.com/). Purified RNA was reverse transcribed using oligo (dT) 20 primers and SuperScript IV reverse transcriptase (ThermoFisher Scientific, https://www.thermofisher.com/). Quantitative RT-PCR was performed using THUNDERBIRD SYBR qPCR Mix (Toyobo, http://www.toyobo-global.com/) and a LightCycler 96 (Roche Diagnostics, https://www.roche.com). Gene expression data were acquired and analysed using LightCycler software. Target gene expression levels were normalized against expression of an internal standard, *UBQ10*.

### Plasmid construction

To generate *pROPGEF4:GFP-ROPGEF4*, *pROPGEF7:YFP-ROPGEF7*, and *pROPGAP4:GEF-ROPGAP4*, the genomic sequence of each gene, including the upstream promoter region, was amplified using PCR and cloned into the pENTR/D-TOPO vector. The *GFP* or *YFP* coding sequence was fused to the 5′ end of the coding sequence using Ligation High II (Toyobo). To generate *pIRX3:ROPGAP3*, 1.5 kbp of the promoter region of *IRX3* was inserted into the NotI site of pENTR/D-TOPO containing the *ROPGAP3* coding sequence. This clone was recombined with the pGWB501 vector. To generate *pLexA:CFP-ROPGAP3*, *p* *LexA:cYFP-ROPGAP3*, and their truncation derivatives, pENTR/D-TOPO carrying full length or truncated *ROPGAP3* were recombined with pER-ECFP-X and pER-xYFP-X. To generate *pLexA:nYFP-ROPGEF4*^*PRONE*^ and *pLexA:cYFP-ROPGEF4*^*PRONE*^, pENTR/D-TOPO carrying *ROPGEF4*^*PRONE*^ was recombined with pER-nYFP-X and pER-cYFP-X^39^. All clones were verified by DNA sequencing. To generate *ROPGEF7RNAi*, the first 400 bp of *ROPGEF7* coding sequence was recombinated with pER-RNAi gateway vector, in which two complementary gateway cassette with an intron spacer was inserted into the ApaI site of pER8 vector.

### Microscopy

An inverted fluorescence microscope (IX83-ZDC, Olympus, https://www.olympus-lifescience.com/) fitted with a confocal unit (CSU-W1, Yokogawa), a cooled CCD camera (ORCA-R2, Hamamatsu Photonics) or an EM-CCD camera (iXon3-888, ANDOR), an UplanSAPO 60× water-immersion objective (NA = 1.2, Olympus), an UplanSAPO 10× objective (NA = 0.4, Olympus), and laser lines set at 458, 488, and 561 nm was used. Images were acquired with MetaMorph software (Molecular Devices). An EM-CCD camera was used for recoding tagRFP-ROPGEF4^PRONE^ and GFP-ROPGAP3 in tobacco leaf epidermis.

To obtain omni-directional views of metaxylem vessels, an Olympus FV3000 inverted confocal microscope (Olympus) equipped with an UPLAN 60× water-immersion objective (NA = 1.2) and a laser line set to 514 nm was used.

### Omni-directional imaging of secondary cell walls

Secondary cell walls of Arabidopsis root cells were stained with propidium iodide, and then z-stack images were acquired using a confocal microscope (FV3000, Olympus). The image stacks were processed and converted into 360° 2-dimensional images using a ImageJ (https://imagej.nih.gov/ij/) plug-in^16^.

### FRET analysis

We calculated the FRET efficiency between CFP-ROPGEF4^PRONE^ and YFP-ROP11 derivatives and between CFP-ROPGEF4^PRONE^ and YFP-ROPGEF4^PRONE^ using the acceptor photo-bleaching method. CFP- and YFP-tagged proteins were expressed in tobacco leaf epidermal cells by the transient gene expression method (see below). Mid plane of the epidermal cells were scanned under FV3000 confocal microscope equipped with an UplanSAPO 60× water-immersion objective (NA = 1.2). Narrow rectangular area of the plasma membrane were photo-bleached with a 514 nm laser at 100% power. Immediately after photo-bleaching, CFP emission was recorded three times with 5 s interval. FRET efficiency was calculated as E = 100 × (A-B)/B, where E indicates FRET efficiency, and B and A indicate the averaged intensity of three images before and after photo-bleaching, respectively. Before the calculation of FRET efficiency, the background intensity was subtracted from the intensity of CFP

### FRAP analysis

FRAP analysis was performed with FV3000 confocal microscope equipped with an UplanSAPO 60× water-immersion objective (NA = 1.2). GFP- or tagRFP-tagged proteins were expressed in tobacco leaf epidermal cells by the transient gene expression method (see below). Mid plane of leaf epidermal cells were scanned, then narrow rectangular area of the plasma membrane at the mid plane were photo-bleached with a 488 nm laser or a 561 nm laser at 100% power for GFP or tagRFP, respectively. Immediately after photo-bleaching, images were scanned every 1 s for 60 s. Recovery rate (%) was calculated as R = 100 × (A-I)/(B-I), where R indicates recovery rate, I, A, and B indicate average intensity of the breached area immediately after bleaching, 60 s after photo-bleaching, and before photo-bleaching, respectively.

### Transient gene expression in *Nicotiana benthamiana*

Transient gene expression in *N. benthamiana* was performed as described previously^17^. *Rhizobium radiobacter* strains GV3101 MP90 harbouring expression constructs were grown on LB media plates with appropriate antibiotics. *R. radiobacter* cells were resuspended in infiltration buffer (10 mM MES (pH 5.7), 10 mM MgCl_2_, 50 mg/L acetosyringone). Equal volumes of *R. radiobacter* suspensions carrying different expression constructs were combined for co-infiltration, and mixed with a suspension of *R. radiobacter* carrying the p19 silencing suppressor in a 1:1 ratio. Mixed suspensions were adjusted to an OD 600 of 1.0. The mixed suspensions were infiltrated into leaves of 3- to 4-week-old *N. benthamiana* plants. The leaf samples were harvested 2 days after infiltration and inoculated with 2 µM oestradiol for subsequent observation for 1 day.

### Quantification of ROPGEF4^PRONE^ domain in the reconstitution assay

To reconstitute ROPGEF4^PRONE^ domains, *pLexA:ROP11*, *pLexA:tag-RFP-ROPGEF4*^*PRONE*^, and *pLexA:ROPGAP3*, were co-introduced into tobacco leaves by the syringe-infiltration with *R. radiobacter*. Two days after infiltration, the leaf samples were harvested and inoculated with 2 µM estradiol. ROPGEF4^PRONE^ domains formed in leaf epidermal cells were observed and recorded using the CSU-W1 system (see above). All images were processed with maximum intensity projection. Subsequently, regions of the ROPGEF4^PRONE^ domains and cell area were manually selected. The numbers of the ROPGEF4^PRONE^ domains were manually counted.

### Quantification of the area of secondary cell wall pits and the distance between pits

To quantify the area of and distance between secondary cell wall pits, the regions and positions of secondary cell wall pits in DIC images were manually selected and analysed using ImageJ. Pit density was calculated as the number of secondary cell wall pits divided by the area of metaxylem vessel cells.

### Statistical analysis

The student’s *t* test, Welch’s *t* test, Wilcoxon rank sum test, and ANOVA with Scheffe test were used to analyse data. Sample sizes (n) and *P*-values are indicated in the figure legends. In scatter plot analyses, Spearman’s rank correlation coefficient was used.

### Mathematical Model

Let us consider the following reaction-diffusion equations on a two-dimensional square space:

S1 $$\begin{array}{rcl}\frac{\partial {u}_{1}}{\partial t} & = & {D}_{1}{{\nabla }}^{2}{u}_{1}+{r}_{6}({u}_{6})-{r}_{1}({u}_{1},{u}_{2},{u}_{3},{F}_{G})\\ \frac{\partial {u}_{2}}{\partial t} & = & {D}_{2}{{\nabla }}^{2}{u}_{2}+{r}_{1}({u}_{1},{u}_{2},{u}_{3},{F}_{G})-{r}_{2}({u}_{2})\\ \frac{\partial {u}_{3}}{\partial t} & = & {D}_{3}{{\nabla }}^{2}{u}_{3}+{r}_{2}({u}_{2})-{r}_{3}({u}_{3})\\ \frac{\partial {u}_{4}}{\partial t} & = & {D}_{4}{{\nabla }}^{2}{u}_{4}+{r}_{3}({u}_{3})-{r}_{4}({u}_{4},{F}_{P}),\\ \frac{\partial {u}_{5}}{\partial t} & = & {D}_{5}{{\nabla }}^{2}{u}_{5}+{r}_{4}({u}_{4},{F}_{P})-{r}_{5}({u}_{5})\\ \frac{\partial {u}_{6}}{\partial t} & = & {D}_{6}{{\nabla }}^{2}{u}_{6}+{r}_{5}({u}_{5})-{r}_{6}({u}_{6})\end{array}$$

where *u*_*i*_(***x***, *t*)s are time-variable spatial distribution of concentrations of ROPs. The concentration of free GEF *F*_*G*_ and free GAP *F*_*P*_ is determined by the following conservation law of GEF and GAP:

S2 $$\begin{array}{c}{T}_{G}={F}_{G}V+{\int }_{0}^{L}{\int }_{0}^{L}dxdy({u}_{2}+{u}_{3})\\ {T}_{P}={F}_{P}V+{\int }_{0}^{L}{\int }_{0}^{L}dxdy({u}_{5}+{u}_{6})\end{array},$$

where *T*_*G*_, *T*_*P*_, *L* and *V* (assumed to be *L*^3^) are constant parameters indicating the total amounts of GEF and GAP, the length of a side of the square space, and the cell volume respectively.

### Fourier Transformation around a stationary state

Suppose that an ordinary differential equation (ODE) system obtained by removal of diffusion terms in (S1) has a positive stationary state ${u}_{i}^{\ast }(i=1,\cdots ,6)$, ${F}_{G}^{\ast }$ and ${F}_{P}^{\ast }$, that should satisfy the following equations:

S3 $$\begin{array}{rcl}{r}_{1}({u}_{1}^{\ast },{u}_{2}^{\ast },{u}_{3}^{\ast },{F}_{G}^{\ast }) & = & {r}_{2}({u}_{2}^{\ast })={r}_{3}({u}_{3}^{\ast })={r}_{4}({u}_{4}^{\ast },{F}_{P}^{\ast })={r}_{5}({u}_{5}^{\ast })={r}_{6}({u}_{6}^{\ast })\\ {T}_{G} & = & {F}_{G}^{\ast }{L}^{3}+({u}_{2}^{\ast }+{u}_{3}^{\ast }){L}^{2}\\ {T}_{P} & = & {F}_{P}^{\ast }{L}^{3}+({u}_{5}^{\ast }+{u}_{6}^{\ast }){L}^{2}\end{array}$$

The perturbations around the stationary state ${u}_{i}^{\ast }$, ${F}_{G}^{\ast }$ and ${F}_{P}^{\ast }$ are:

S4 $${u}_{i}({\boldsymbol{x}},t)={u}_{i}^{\ast }+\delta {u}_{i}({\boldsymbol{x}},t),\,{F}_{G}({\boldsymbol{x}},t)={F}_{G}^{\ast }+\delta {F}_{G}({\boldsymbol{x}},t),{F}_{P}={F}_{P}^{\ast }+\delta {F}_{P}({\boldsymbol{x}},t).$$

The differential equations for the perturbations are written as:

S5 $$\begin{array}{rcl}\frac{\partial \delta {u}_{1}}{\partial t} & = & {D}_{1}{{\nabla }}^{2}\delta {u}_{1}+{\frac{\partial {r}_{6}}{\partial {u}_{6}}|}_{\ast }\delta {u}_{6}-{\frac{\partial {r}_{1}}{\partial {u}_{1}}|}_{\ast }\delta {u}_{1}-{\frac{\partial {r}_{1}}{\partial {u}_{2}}|}_{\ast }\delta {u}_{2}-{\frac{\partial {r}_{1}}{\partial {u}_{3}}|}_{\ast }\delta {u}_{3}-{\frac{\partial {r}_{1}}{\partial {F}_{G}}|}_{\ast }\delta {F}_{G}\\ \frac{\partial \delta {u}_{2}}{\partial t} & = & {D}_{2}{{\nabla }}^{2}\delta {u}_{2}+{\frac{\partial {r}_{1}}{\partial {u}_{1}}|}_{\ast }\delta {u}_{1}+{\frac{\partial {r}_{1}}{\partial {u}_{2}}|}_{\ast }\delta {u}_{2}+{\frac{\partial {r}_{1}}{\partial {u}_{3}}|}_{\ast }\delta {u}_{3}+{\frac{\partial {r}_{1}}{\partial {F}_{G}}|}_{\ast }\delta {F}_{G}-{\frac{\partial {r}_{2}}{\partial {u}_{2}}|}_{\ast }\delta {u}_{2}\\ \frac{\partial \delta {u}_{3}}{\partial t} & = & {D}_{3}{{\nabla }}^{2}\delta {u}_{3}+{\frac{\partial {r}_{2}}{\partial {u}_{2}}|}_{\ast }\delta {u}_{2}-{\frac{\partial {r}_{3}}{\partial {u}_{3}}|}_{\ast }\delta {u}_{3}\\ \frac{\partial \delta {u}_{4}}{\partial t} & = & {D}_{4}{{\nabla }}^{2}\delta {u}_{4}+{\frac{\partial {r}_{3}}{\partial {u}_{3}}|}_{\ast }\delta {u}_{3}-{\frac{\partial {r}_{4}}{\partial {u}_{4}}|}_{\ast }\delta {u}_{4}-{\frac{\partial {r}_{4}}{\partial {F}_{P}}|}_{\ast }\delta {F}_{P}\\ \frac{\partial \delta {u}_{5}}{\partial t} & = & {D}_{5}{{\nabla }}^{2}\delta {u}_{5}+{\frac{\partial {r}_{4}}{\partial {u}_{4}}|}_{\ast }\delta {u}_{4}+{\frac{\partial {r}_{4}}{\partial {F}_{P}}|}_{\ast }\delta {F}_{P}-{\frac{\partial {r}_{5}}{\partial {u}_{5}}|}_{\ast }\delta {u}_{5}\\ \frac{\partial \delta {u}_{6}}{\partial t} & = & {D}_{6}{{\nabla }}^{2}\delta {u}_{6}+{\frac{\partial {r}_{5}}{\partial {u}_{5}}|}_{\ast }\delta {u}_{5}-{\frac{\partial {r}_{6}}{\partial {u}_{6}}|}_{\ast }\delta {u}_{6}\end{array},$$

where the partial derivatives are evaluated at the stationary state ${u}_{i}^{\ast }$, ${F}_{G}^{\ast }$, ${F}_{P}^{\ast }$. From the conservation laws, the perturbations of *F*_*G*_ and *F*_*P*_ are given by

S6 $$\begin{array}{c}\delta {F}_{G}=-\,\frac{1}{V}{\int }_{0}^{L}{\int }_{0}^{L}dxdy(\delta {u}_{2}+\delta {u}_{3})\\ \delta {F}_{P}=-\,\frac{1}{V}{\int }_{0}^{L}{\int }_{0}^{L}dxdy(\delta {u}_{5}+\delta {u}_{6})\end{array}.$$

We take Fourier transformation:

S7 $$\begin{array}{c}\delta {\tilde{u}}_{i}^{m}=\frac{1}{{L}^{2}}{\int }_{0}^{L}{\int }_{0}^{L}dxdy{e}^{-i{\boldsymbol{m}}\cdot {\boldsymbol{x}}}\delta {u}_{i}({\boldsymbol{x}},t)\\ \delta {\tilde{F}}_{G}^{m}=-\frac{1}{V}{\delta }_{{n}_{x},0}{\delta }_{{n}_{y},0}(\delta {\tilde{u}}_{2}^{0}+\delta {\tilde{u}}_{3}^{0})\\ \delta {\tilde{F}}_{P}^{m}=-\frac{1}{V}{\delta }_{{n}_{x},0}{\delta }_{{n}_{y},0}(\delta {\tilde{u}}_{5}^{0}+\delta {\tilde{u}}_{6}^{0})\end{array},$$

where ${\boldsymbol{m}}=({m}_{x},{m}_{y})=(\frac{2\pi {n}_{x}}{L},\frac{2\pi {n}_{y}}{L}),({n}_{x},{n}_{y}\in {\mathbb{N}})$, $m=\sqrt{{m}_{x}^{2}+{m}_{y}^{2}}$, and *δ*_*i*,*j*_ indicates Kronecker delta:

$${\delta }_{i,j}=\{\begin{array}{c}1\,(i=j)\\ 0\,(i\ne j)\end{array}.$$

Then the following equations must be hold.

S8 $$\begin{array}{c}\frac{\partial \delta {\tilde{u}}_{1}^{m}}{\partial t}=-{m}^{2}{D}_{1}\delta {\tilde{u}}_{1}^{m}+{\frac{\partial {r}_{6}}{\partial {u}_{6}}|}_{\ast }\delta {\tilde{{u}_{6}}}^{m}-{\frac{\partial {r}_{1}}{\partial {u}_{1}}|}_{\ast }\delta {\tilde{u}}_{1}^{m}-{\frac{\partial {r}_{1}}{\partial {u}_{2}}|}_{\ast }\delta {\tilde{u}}_{2}^{m}-{\frac{\partial {r}_{1}}{\partial {u}_{3}}|}_{\ast }\delta {\tilde{u}}_{3}^{m}-{\frac{\partial {r}_{1}}{\partial {F}_{G}}|}_{\ast }\delta {\tilde{F}}_{G}^{m}\\ \frac{\partial \delta {\tilde{u}}_{2}^{m}}{\partial t}=-{m}^{2}{D}_{2}\delta {\tilde{u}}_{2}^{m}+{\frac{\partial {r}_{1}}{\partial {u}_{1}}|}_{\ast }\delta {\tilde{u}}_{1}^{m}+{\frac{\partial {r}_{1}}{\partial {u}_{2}}|}_{\ast }\delta {\tilde{u}}_{2}^{m}+{\frac{\partial {r}_{1}}{\partial {u}_{3}}|}_{\ast }\delta {\tilde{u}}_{3}^{m}+{\frac{\partial {r}_{1}}{\partial {F}_{G}}|}_{\ast }\delta {\tilde{F}}_{G}^{m}-{\frac{\partial {r}_{2}}{\partial {u}_{2}}|}_{\ast }\delta {\tilde{u}}_{2}^{m}\\ \frac{\partial \delta {\tilde{u}}_{2}^{m}}{\partial t}=-{m}^{2}{D}_{2}\delta {\tilde{u}}_{2}^{m}+{\frac{\partial {r}_{1}}{\partial {u}_{1}}|}_{\ast }\delta {\tilde{u}}_{1}^{m}+{\frac{\partial {r}_{1}}{\partial {u}_{2}}|}_{\ast }\delta {\tilde{u}}_{2}^{m}+{\frac{\partial {r}_{1}}{\partial {u}_{3}}|}_{\ast }\delta {\tilde{u}}_{3}^{m}+{\frac{\partial {r}_{1}}{\partial {F}_{G}}|}_{\ast }\delta {\tilde{F}}_{G}^{m}-{\frac{\partial {r}_{2}}{\partial {u}_{2}}|}_{\ast }\delta {\tilde{u}}_{2}^{m}\\ \frac{\partial \delta {\tilde{u}}_{4}^{m}}{\partial t}=-{m}^{2}{D}_{4}\delta {\tilde{u}}_{4}^{m}+{\frac{\partial {r}_{3}}{\partial {u}_{3}}|}_{\ast }\delta {\tilde{u}}_{3}^{m}-{\frac{\partial {r}_{4}}{\partial {u}_{4}}|}_{\ast }\delta {\tilde{u}}_{4}^{m}-{\frac{\partial {r}_{4}}{\partial {F}_{P}}|}_{\ast }\delta {\tilde{F}}_{P}^{m}\\ \frac{\partial \delta {\tilde{u}}_{5}^{m}}{\partial t}=-{m}^{2}{D}_{5}\delta {\tilde{u}}_{5}^{m}+{\frac{\partial {r}_{4}}{\partial {u}_{4}}|}_{\ast }\delta {\tilde{u}}_{4}^{m}+{\frac{\partial {r}_{4}}{\partial {F}_{P}}|}_{\ast }\delta {\tilde{F}}_{P}^{m}-{\frac{\partial {r}_{5}}{\partial {u}_{5}}|}_{\ast }\delta {\tilde{u}}_{5}^{m}\\ \frac{\partial \delta {\tilde{u}}_{6}^{m}}{\partial t}=-{m}^{2}{D}_{6}\delta {\tilde{u}}_{6}^{m}+{\frac{\partial {r}_{5}}{\partial {u}_{5}}|}_{\ast }\delta {\tilde{u}}_{5}^{m}-{\frac{\partial {r}_{5}}{\partial {u}_{6}}|}_{\ast }\delta {\tilde{u}}_{6}^{m}\end{array}$$

The linearized dynamics in the Fourier space is written separately for the mode *m* is 0 and positive. For the Fourier mode *m* = 0,

S9 $$\begin{array}{l}\frac{d}{dt}(\begin{array}{c}\begin{array}{c}\begin{array}{c}\begin{array}{c}\delta {\tilde{u}}_{1}^{0}\\ \delta {\tilde{u}}_{2}^{0}\\ \delta {\tilde{u}}_{3}^{0}\end{array}\\ \delta {\tilde{u}}_{4}^{0}\end{array}\\ \delta {\tilde{u}}_{5}^{0}\end{array}\\ \delta {\tilde{u}}_{6}^{0}\end{array})\\ =(\begin{array}{cccccc}-{\frac{\partial {r}_{1}}{\partial {u}_{1}}|}_{\ast } & -{\frac{\partial {r}_{1}}{\partial {u}_{2}}|}_{\ast }-\frac{1}{V}{\frac{\partial {r}_{1}}{\partial {F}_{G}}|}_{\ast } & -{\frac{\partial {r}_{1}}{\partial {u}_{3}}|}_{\ast }-\frac{1}{V}{\frac{\partial {r}_{1}}{\partial {F}_{G}}|}_{\ast } & 0 & 0 & {\frac{\partial {r}_{6}}{\partial {u}_{6}}|}_{\ast }\\ {\frac{\partial {r}_{1}}{\partial {u}_{1}}|}_{\ast } & -{\frac{\partial {r}_{2}}{\partial {u}_{2}}|}_{\ast }+{\frac{\partial {r}_{1}}{\partial {u}_{2}}|}_{\ast }+\frac{1}{V}{\frac{\partial {r}_{1}}{\partial {F}_{G}}|}_{\ast } & {\frac{\partial {r}_{1}}{\partial {u}_{3}}|}_{\ast }+\frac{1}{V}{\frac{\partial {r}_{1}}{\partial {F}_{G}}|}_{\ast } & 0 & 0 & 0\\ 0 & {\frac{\partial {r}_{2}}{\partial {u}_{2}}|}_{\ast } & -{\frac{\partial {r}_{3}}{\partial {u}_{3}}|}_{\ast } & 0 & 0 & 0\\ 0 & 0 & {\frac{\partial {r}_{3}}{\partial {u}_{3}}|}_{\ast } & -{\frac{\partial {r}_{4}}{\partial {u}_{4}}|}_{\ast } & -\frac{1}{V}{\frac{\partial {r}_{4}}{\partial {F}_{P}}|}_{\ast } & -\frac{1}{V}{\frac{\partial {r}_{4}}{\partial {F}_{P}}|}_{\ast }\\ 0 & 0 & 0 & {\frac{\partial {r}_{4}}{\partial {u}_{4}}|}_{\ast } & -{\frac{\partial {r}_{5}}{\partial {u}_{5}}|}_{\ast }+\frac{1}{V}{\frac{\partial {r}_{4}}{\partial {F}_{P}}|}_{\ast } & \frac{1}{V}{\frac{\partial {r}_{4}}{\partial {F}_{P}}|}_{\ast }\\ 0 & 0 & 0 & 0 & {\frac{\partial {r}_{5}}{\partial {u}_{5}}|}_{\ast } & -{\frac{\partial {r}_{6}}{\partial {u}_{6}}|}_{\ast }\end{array})\,(\begin{array}{c}\begin{array}{c}\begin{array}{c}\begin{array}{c}\delta {\tilde{u}}_{1}^{0}\\ \delta {\tilde{u}}_{2}^{0}\\ \delta {\tilde{u}}_{3}^{0}\end{array}\\ \delta {\tilde{u}}_{4}^{0}\end{array}\\ \delta {\tilde{u}}_{5}^{0}\end{array}\\ \delta {\tilde{u}}_{6}^{0}\end{array})\\ \equiv (\begin{array}{cccccc}-{K}_{1} & -{K}_{7}+{p}_{1} & -{K}_{8}+{p}_{1} & 0 & 0 & {K}_{6}\\ {K}_{1} & -{K}_{2}+{K}_{7}-{p}_{1} & {K}_{8}-{p}_{1} & 0 & 0 & 0\\ 0 & {K}_{2} & -{K}_{3} & 0 & 0 & 0\\ 0 & 0 & {K}_{3} & -{K}_{4} & {p}_{2} & {p}_{2}\\ 0 & 0 & 0 & {K}_{4} & -{K}_{5}-{p}_{2} & -{p}_{2}\\ 0 & 0 & 0 & 0 & {K}_{5} & -{K}_{6}\end{array})\,(\begin{array}{c}\begin{array}{c}\begin{array}{c}\begin{array}{c}\delta {\tilde{u}}_{1}^{0}\\ \delta {\tilde{u}}_{2}^{0}\\ \delta {\tilde{u}}_{3}^{0}\end{array}\\ \delta {\tilde{u}}_{4}^{0}\end{array}\\ \delta {\tilde{u}}_{5}^{0}\end{array}\\ \delta {\tilde{u}}_{6}^{0}\end{array})\end{array}$$

where the parameters *K*_1_ ~ *K*_8_,*p*_1_,*p*_2_ are defined as:

S10 $$\begin{array}{rcl}{K}_{1}={\frac{\partial {r}_{1}}{\partial {u}_{1}}|}_{\ast },{K}_{2} & = & {\frac{\partial {r}_{2}}{\partial {u}_{2}}|}_{\ast },{K}_{3}={\frac{\partial {r}_{3}}{\partial {u}_{3}}|}_{\ast },{K}_{4}={\frac{\partial {r}_{4}}{\partial {u}_{4}}|}_{\ast },\\ {K}_{5} & = & {\frac{\partial {r}_{5}}{\partial {u}_{5}}|}_{\ast },{K}_{6}={\frac{\partial {r}_{6}}{\partial {u}_{6}}|}_{\ast },{K}_{7}={\frac{\partial {r}_{1}}{\partial {u}_{2}}|}_{\ast },{K}_{8}={\frac{\partial {r}_{1}}{\partial {u}_{3}}|}_{\ast }\\ {p}_{1} & = & -\frac{1}{V}{\frac{\partial {r}_{1}}{\partial {F}_{G}}|}_{\ast }\\ {p}_{2} & = & -\frac{1}{V}{\frac{\partial {r}_{4}}{\partial {F}_{P}}|}_{\ast }.\end{array}.$$

For the Fourier mode $m > 0$,

S11 $$\begin{array}{c}\frac{d}{dt}(\begin{array}{c}\begin{array}{c}\begin{array}{c}\begin{array}{c}\delta {\tilde{u}}_{1}^{m}\\ \delta {\tilde{u}}_{2}^{m}\\ \delta {\tilde{u}}_{3}^{m}\end{array}\\ \delta {\tilde{u}}_{4}^{m}\end{array}\\ \delta {\tilde{u}}_{5}^{m}\end{array}\\ \delta {\tilde{u}}_{6}^{m}\end{array})\\ =(\begin{array}{cccccc}-{\frac{\partial {r}_{1}}{\partial {u}_{1}}|}_{\ast }-{m}^{2}{D}_{1} & -{\frac{\partial {r}_{1}}{\partial {u}_{2}}|}_{\ast } & -{\frac{\partial {r}_{1}}{\partial {u}_{3}}|}_{\ast } & 0 & 0 & {\frac{\partial {r}_{6}}{\partial {u}_{6}}|}_{\ast }\\ {\frac{\partial {r}_{1}}{\partial {u}_{1}}|}_{\ast } & -{\frac{\partial {r}_{2}}{\partial {u}_{2}}|}_{\ast }+{\frac{\partial {r}_{1}}{\partial {u}_{2}}|}_{\ast }-{m}^{2}{D}_{2} & {\frac{\partial {r}_{1}}{\partial {u}_{3}}|}_{\ast } & 0 & 0 & 0\\ 0 & {\frac{\partial {r}_{2}}{\partial {u}_{2}}|}_{\ast } & -{\frac{\partial {r}_{3}}{\partial {u}_{3}}|}_{\ast }-{m}^{2}{D}_{3} & 0 & 0 & 0\\ 0 & 0 & {\frac{\partial {r}_{3}}{\partial {u}_{3}}|}_{\ast } & -{\frac{\partial {r}_{4}}{\partial {u}_{4}}|}_{\ast }-{m}^{2}{D}_{4} & 0 & 0\\ 0 & 0 & 0 & {\frac{\partial {r}_{4}}{\partial {u}_{4}}|}_{\ast } & -{\frac{\partial {r}_{5}}{\partial {u}_{5}}|}_{\ast }-{m}^{2}{D}_{5} & 0\\ 0 & 0 & 0 & 0 & {\frac{\partial {r}_{5}}{\partial {u}_{5}}|}_{\ast } & -{\frac{\partial {r}_{6}}{\partial {u}_{6}}|}_{\ast }-{m}^{2}{D}_{6}\end{array})(\begin{array}{c}\begin{array}{c}\begin{array}{c}\begin{array}{c}\delta {\tilde{u}}_{1}^{m}\\ \delta {\tilde{u}}_{2}^{m}\\ \delta {\tilde{u}}_{3}^{m}\end{array}\\ \delta {\tilde{u}}_{4}^{m}\end{array}\\ \delta {\tilde{u}}_{5}^{m}\end{array}\\ \delta {\tilde{u}}_{6}^{m}\end{array})\\ =(\begin{array}{cccccc}-{K}_{1}-{m}^{2}{D}_{1} & -{K}_{7} & -{K}_{8} & 0 & 0 & {K}_{6}\\ {K}_{1} & -{K}_{2}+{K}_{7}-{m}^{2}{D}_{2} & {K}_{8} & 0 & 0 & 0\\ 0 & {K}_{2} & -{K}_{3}-{m}^{2}{D}_{3} & 0 & 0 & 0\\ 0 & 0 & {K}_{3} & -{K}_{4}-{m}^{2}{D}_{4} & 0 & 0\\ 0 & 0 & 0 & {K}_{4} & -{K}_{5}-{m}^{2}{D}_{5} & 0\\ 0 & 0 & 0 & 0 & {K}_{5} & -{K}_{6}-{m}^{2}{D}_{6}\end{array})(\begin{array}{c}\begin{array}{c}\begin{array}{c}\begin{array}{c}\delta {\tilde{u}}_{1}^{m}\\ \delta {\tilde{u}}_{2}^{m}\\ \delta {\tilde{u}}_{3}^{m}\end{array}\\ \delta {\tilde{u}}_{4}^{m}\end{array}\\ \delta {\tilde{u}}_{5}^{m}\end{array}\\ \delta {\tilde{u}}_{6}^{m}\end{array})\end{array}$$

The biological evidences require the positive values for the parameters ${K}_{i} > 0$, (*i* = 1, …, 6), non-negative values for ${K}_{i}\ge 0$ (*i* = 7, 8), and non-positive values for $\,{p}_{1}\le 0$,$\,{p}_{2}\le 0$.

### Mathematical analysis

The condition of a reaction-diffusion system for periodic pattern formation was given as Turing’s instability, which consists of two parts: (i) equilibrium of the linearized ODE system (S9) is stable; and (ii) homogeneous distribution of the stationary state is unstable, i.e., the linearized PDE (S11) is unstable for some values of *m*. The stability of the linearized system is determined by the Routh-Hurwitz criterion (see below) from the linearized matrix ***J*** or its correspondence ***J***−*m*^2^***D*** (Murray, 2002). In a model with *n* variables, the condition of stability is given as intersections of *n* inequalities. We analysed the stability condition symbolically from the distribution of zero and nonzero entries in the Jacobian matrixes ***J*** and ***J*** − *m*^2^***D***^40^. We call such symbolic analysis “structural analysis”. By the structural analyses, we discuss conditions of structures of the reaction network to satisfy the Turing instability without depending on details of nonlinear functions of the systems.

Three different versions of the models were compared: (A) Closed-circuit model without regulations (*p*_1_ = 0, *p*_2_ = 0; *K*_7_ = *K*_8_ = 0); (B) Inhibition from conserved quantities (${p}_{1} < 0$, ${p}_{2} < 0$; *K*_7_ = *K*_8_ = 0); and (C) Positive feedback from dimerization (*p*_1_ = 0, *p*_2_ = 0; ${K}_{7} > 0$, ${K}_{8} > 0$). The symbolic formula of six inequalities for the Turing condition was determined and analysed based on Routh-Hurwitz criterion via Mathematica. The closed-circuit model (A) and the inhibition model (B) never show Turing instability from their structure. The positive feedback model (C) can represent the Turing instability. The results give the structural conditions of the reaction network required for Turing instability. Positive feedback is necessary while the conservation law does not contribute to the Turing instability.

### Routh-Hurwitz criterion

The Routh-Hurwitz criterion, which gives a necessary and sufficient condition for the stability of a linear dynamical system, is described as follows. Given the characteristic polynomial, $P(\lambda )={\lambda }^{n}+{a}_{1}{\lambda }^{n-1}+\cdots +{a}_{n-1}\lambda +{a}_{n}$ with all real coefficients *a*_*i*_, *i* = 1, …*n*, the *n* Hurwitz matrices are defined using the coefficients *a*_*i*_ of the characteristic polynomial:

S12 $${H}_{1}={a}_{1},{H}_{2}=(\begin{array}{cc}{a}_{1} & 1\\ {a}_{3} & {a}_{2}\end{array}),{H}_{3}=(\begin{array}{ccc}{a}_{1} & 1 & 0\\ {a}_{3} & {a}_{2} & {a}_{1}\\ {a}_{5} & {a}_{4} & {a}_{3}\end{array})$$

and

S13 $${H}_{n}=(\begin{array}{ccc}{a}_{1} & 1 & \begin{array}{ccc}0 & 0 & \begin{array}{cc}\cdots  & 0\end{array}\end{array}\\ {a}_{3} & {a}_{2} & \begin{array}{ccc}{a}_{1} & 1 & \begin{array}{cc}\cdots  & 0\end{array}\end{array}\\ \begin{array}{c}{a}_{5}\\ \vdots \\ 0\end{array} & \begin{array}{c}{a}_{4}\\ \vdots \\ 0\end{array} & \begin{array}{c}\begin{array}{ccc}{a}_{3} & {a}_{2} & \begin{array}{cc}\cdots  & 0\end{array}\end{array}\\ \begin{array}{ccc}\vdots  & \vdots  & \begin{array}{cc}\ddots  & \vdots \end{array}\end{array}\\ \begin{array}{ccc}0 & 0 & \begin{array}{cc}\cdots  & {a}_{n}\end{array}\end{array}\end{array}\end{array}),$$

where *a*_*j*_ = 0 for $j > n$. Then, all of the roots of the polynomial equation *P*(*λ*) = 0 are negative or have negative real part, if and only if the determinants of all Hurwitz matrices are positive:

S14 $${\rm{\det }}\,{H}_{j} > 0,j=1,2,\cdots ,n.$$

### Numerical analysis

Using the positive feedback model (C), which can satisfy the Turing instability condition structurally, the qualitative condition of 14 parameters (${K}_{1} \sim {K}_{8}$ and ${D}_{1} \sim {D}_{6}$) was examined. We generated 10^8^ sets of parameters randomly using uniform distributions of ${K}_{1} \sim {K}_{8}\in (0,10]$ and ${D}_{1} \sim {D}_{6}\in (0,{10}^{2}]$. Among them, 580,362 parameter sets satisfied the Turing condition qualitatively. The probability *P*(*K*_*i*_)*dK*_*i*_ for $i=1 \sim 8$ and *P*(*D*_*i*_)*dD*_*i*_ for $i=1 \sim 6$ are shown in Fig. 3B and C. The obtained values of parameters *K*_1_ and *K*_7_ were biased larger and that of *K*_2_ was biased lower, respectively. The obtained values of *D*_1_ was biased larger, and that of *D*_3_ was lower. Especially, the obtained value of the parameter *D*_2_ was significantly small compared with the other diffusion coefficients.

### Numerical simulation

To calculate the dynamics of the reaction-diffusion systems of the model including all feedback regulations (${p}_{1} < 0$, ${p}_{2} < 0$; ${K}_{7} > 0$, ${K}_{8} > 0$), we adopt the following nonlinear reaction system:

S15 $$\begin{array}{rcl}\frac{\partial {u}_{1}}{\partial t} & = & {D}_{1}{{\nabla }}^{2}{u}_{1}+{k}_{6}{u}_{6}-\frac{a{F}_{G}{u}_{1}{({u}_{2}+c{u}_{3})}^{n}}{{K}_{m}+{({u}_{2}+c{u}_{3})}^{n}}\\ \frac{\partial {u}_{2}}{\partial t} & = & {D}_{2}{{\nabla }}^{2}{u}_{2}+\frac{a{F}_{G}{u}_{1}{({u}_{2}+c{u}_{3})}^{n}}{{K}_{m}+{({u}_{2}+c{u}_{3})}^{n}}-{k}_{2}{u}_{2}\\ \frac{\partial {u}_{3}}{\partial t} & = & {D}_{3}{{\nabla }}^{2}{u}_{3}+{k}_{2}{u}_{2}-{k}_{3}{u}_{3}\\ \frac{\partial {u}_{4}}{\partial t} & = & {D}_{4}{{\nabla }}^{2}{u}_{4}+{k}_{3}{u}_{3}-{k}_{4}{F}_{P}{u}_{4}\\ \frac{\partial {u}_{5}}{\partial t} & = & {D}_{5}{{\nabla }}^{2}{u}_{5}+{k}_{4}{F}_{P}{u}_{4}-{k}_{5}{u}_{5}\\ \frac{\partial {u}_{6}}{\partial t} & = & {D}_{6}{{\nabla }}^{2}{u}_{6}+{k}_{5}{u}_{5}-{k}_{6}{u}_{6}\end{array}$$

with the Hill coefficient *n* = 5. The following relations connects the general formulation shown in (S1) and the above specific choices of functions (S15):

S16 $$\begin{array}{rcl}{r}_{1}({u}_{1},{u}_{2},{u}_{3},{F}_{G}) & = & \frac{a{F}_{G}{u}_{1}{({u}_{2}+c{u}_{3})}^{n}}{{K}_{m}+{({u}_{2}+c{u}_{3})}^{n}}\\ {r}_{2}({u}_{2}) & = & {k}_{2}{u}_{2}\\ {r}_{3}({u}_{3}) & = & {k}_{3}{u}_{3}\\ {r}_{4}({u}_{4},{F}_{P}) & = & {k}_{4}{F}_{P}{u}_{4}\\ {r}_{5}({u}_{5}) & = & {k}_{5}{u}_{5}\\ {r}_{6}({u}_{6}) & = & {k}_{6}{u}_{6}\end{array}.$$

The parameter values and initial states are selected from randomly determined sets, that satisfy the Turing instability condition quantitatively. The chosen parameters are: *a* = 57.113932, *K*_*m*_ = 3.097872, *c* = 0.658876, *k*_2_ = 2.261496, *k*_3_ = 5.751476, *k*_4_ = 3.324223, *k*_5_ = 2.002325, *k*_6_ = 7.099055, *D*_1_ = 42.373001, *D*_2_ = 2.107038, *D*_3_ = 54.475096, *D*_4_ = 52.561383, *D*_5_ = 52.796738, and *D*_6_ = 71.697538. At the initial state, concentrations of *u*_*i*_s were given homogeneously as ${\bar{u}}_{1}^{0}=0.18$, ${\bar{u}}_{2}^{0}=0.57$, ${\bar{u}}_{3}^{0}=0.22$, ${\bar{u}}_{4}^{0}=0.15$, ${\bar{u}}_{5}^{0}=0.65$, and ${\bar{u}}_{6}^{0}=0.18$. with small fluctuations. The other parameters are given as *T*_*G*_ = 105*L*^2^, *T*_*P*_ = 105*L*^2^.

Alternative Direction Implicit Method (ADI method) (W. H. Press, S. A. Teukolsky, W. T. Vetterling, B. P. Flannery (2007). “Section 20.3.3. Operator Splitting Methods Generally”. *Numerical Recipes: The Art of Scientific Computing* (3rd ed.). New York: Cambridge University Press.) was used for numerical calculations with a small time-step (${\rm{\Delta }}t=0.01$). For numerical calculations, a square lattice including 100×100 sites with a periodic boundary condition was used. The time evolution of *u*_*i*_ patterns in the numerical calculation is shown in Fig. 4E (times *t* =  0, 5 and 100).

## Electronic supplementary material

Supplementary Information

Movie S1
