# Supplementary material for: A Rho-based reaction-diffusion system governs cell wall patterning in metaxylem vessels
Source: Sci Rep. 2018 Aug 1;8:11542. doi: 10.1038/s41598-018-29543-y (PMC6070580; doi:10.1038/s41598-018-29543-y)
Supplement: Supplementary file 1 — Supplementary Information [file 41598_2018_29543_MOESM1_ESM.pdf]

A Rho-based reaction-diffusion system governs cell wall patterning in metaxylem vessels

Yoshinobu Nagashima, Satoru Tsugawa, Atsushi Mochizuki, Takema Sasaki, Hiroo Fukuda, Yoshihisa Oda

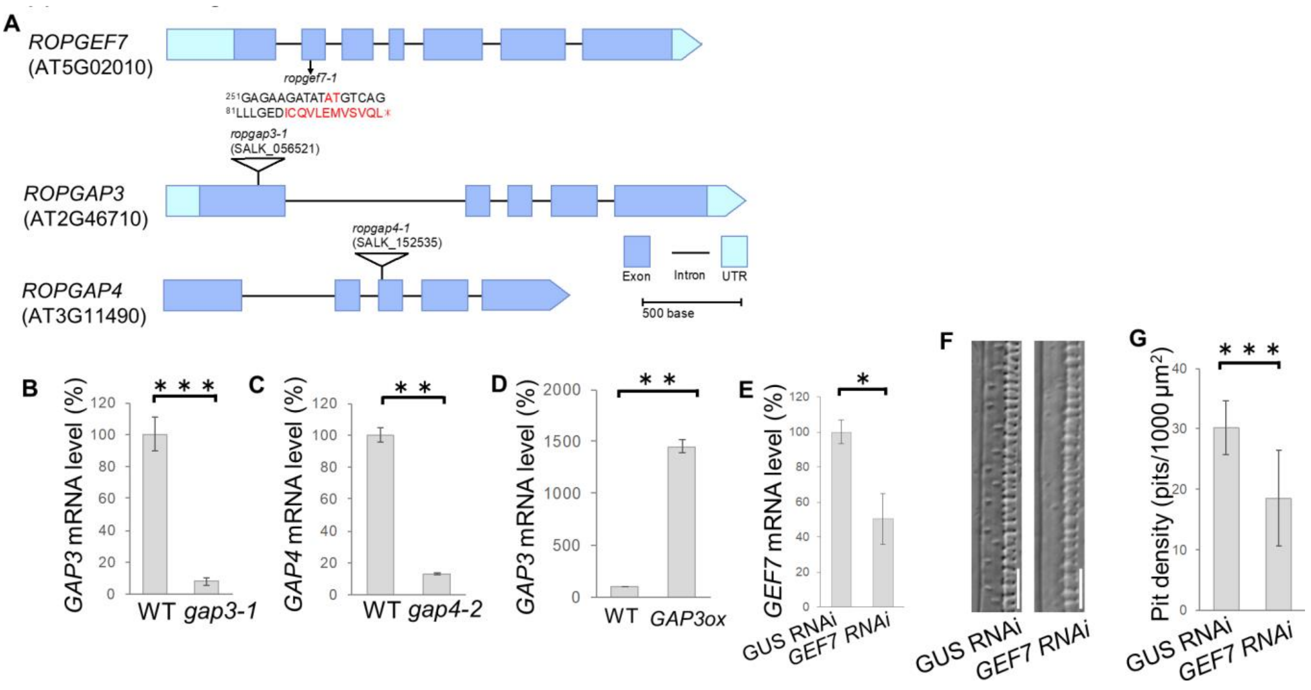

Supplemental Figure S1. *ropgef* and *ropgap* mutants

(A) Structure of *ROPGEF7(GEF7), ROPGAP3(GAP3) and ROPGAP4(GAP4)*. The *ropgef7-1* mutant contains an AT-insertion in the second exon. Red characters indicate the additional nucleotides and the amino acid sequence altered by frame shift.

(B and D) *ROPGAP3* mRNA levels in wild-type (WT) and *ropgap3-1* (B) or *ROPGAP3ox* (*GAP3ox*) (D) plants. Data are means ± SD (n = 3); ***P < 0.001; student's t test (B); **P < 0.01; Welch's t test (D).

(C) *ROPGAP4* mRNA levels in WT and *ropgap4-2* plants. Data are means ± SD (n = 3). **P < 0.01; Welch's t test.

(E-G) *ROPGEF7* mRNA levels (E), DIC of xylem vessels (F), and density of secondary cell wall pits (G) in *GUS RNAi* and *GEF7 RNAi* plants. Data are means ± SD (n = 3 (E) and 12(G)); *P < 0.05;***P < 0.001 student's t test; Scale bars = 10 µm (F).

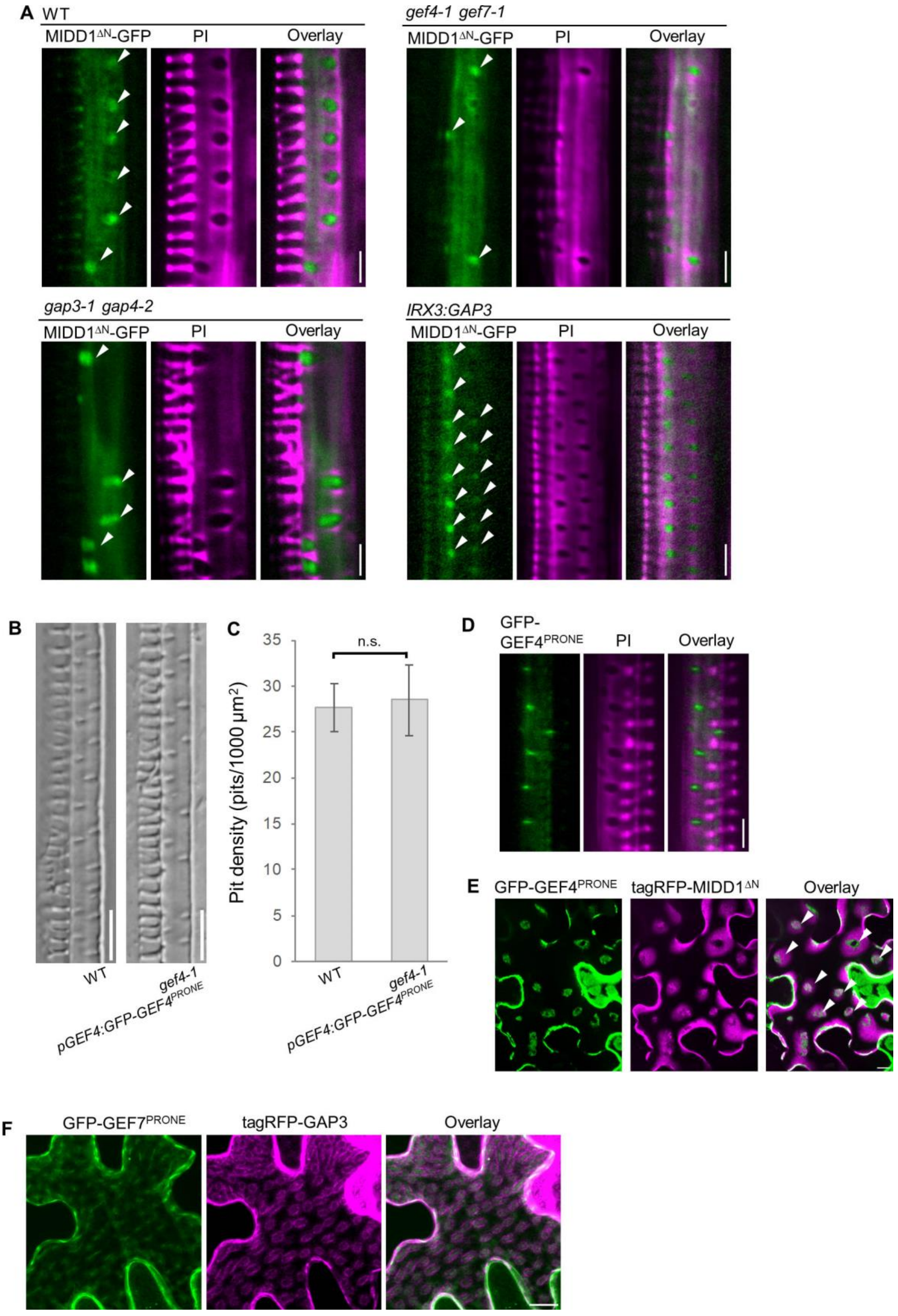

Supplemental Figure S2. Visualization of ROP-activated domains.

(A) Xylem vessel cells in the roots of wild type (WT), *ropgef4-1 ropgef7-1* (*gef4-1 gef7-1*), *ropgap3-1 ropgap4-2* (*gap3-1 gap4-2*), and *pIRX:ROPGAP3* (*IRX3:GAP3*) plants harbouring *pMIDD1:MIDD1* $^{\triangle N}$*-GFP*. ROP activated domains are labelled with MIDD1$^{\triangle \mathrm{N}}$-GFP (arrowheads). Cell walls are stained with PI.

(B) DIC of metaxylem vessels in the roots of wild-type plants (WT) and *ropgef4-1 pROPGEF4:GFP-ROPGEF4*^{PRONE} plants (pGEF4:GFP-GEF$^{\mathrm{4PRONE}}$).

(C) Density of secondary cell wall pits in WT and *ropgef4-1 pROPGEF4:GFP-ROPGEF4*^{PRONE} plants. Data are means ± SD (n = 12 plants). n.s. means not significant; student's *t* test.

(D) Localization of GFP-ROPGEF4^{PRONE} in *ropgef4-1 pROPGEF4:GFP-ROPGEF4*^{PRONE} plants. Cell walls are stained with propidium iodide (PI).

(E) Reconstruction of ROP-activated domains in tobacco leaf epidermal cells. *pLexA:GFP-ROPGEF4* ^{PRONE}, *pLexA:ROP11*, and *pLexA:ROPGAP3* were co-introduced to leaves of *N. benthamiana* along with *pLexA:tagRFP-MIDD1*$^{\triangle N}$, a marker for active ROP11. Arrowheads indicate ROP-activated domains marked with tagRFP-MIDD1$^{\triangle \mathrm{N}}$, formed just around ROPGEF4^{PRONE} domains.

(F) Reconstruction of ROPGEF7^{PRONE} domains in tobacco leaf epidermal cells. *pLexA:GFP-ROPGEF7* ^{PRONE} was co-introduced to leaves of *N. benthamiana* along with *pLexA:ROP*2 and *pLexA:tagRFP-ROPGAP3*.

Scale bars = 5 µm (C and E) and 10 µm (A, D, and F).

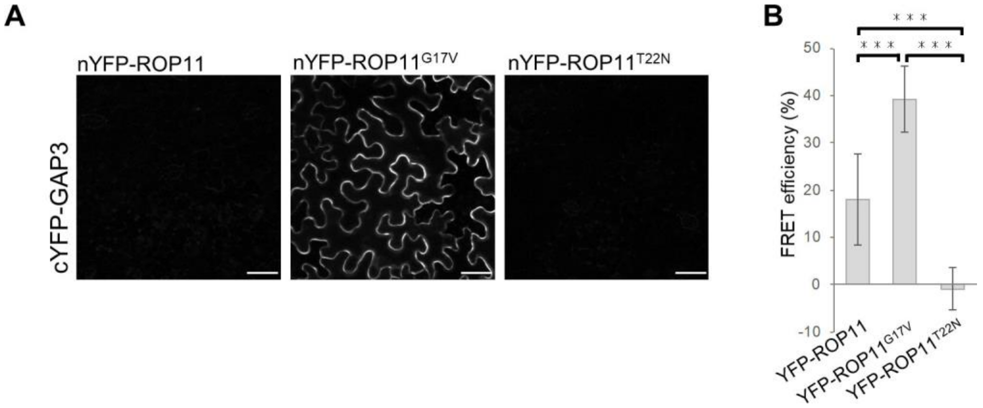

Supplemental Figure S3. ROPGAP3 preferentially interacts with GTP-ROP11 in vivo

(A) BiFC assay between cYFP-ROPGAP3 and nYFP-ROP11 derivatives. Note that YFP signal was detected only when cYFP-ROPGAP3 was co-expressed with YFP-ROP11^{G17V}. Scale bars = 50 µm.

(B) FRET efficiency between CFP-ROPGAP3 and YFP-ROP11 derivatives. Data are means ± SD (n = 15). ***$P < 0.001$; ANOVA with Scheffe’s test.

Supplemental Table S1. Turing instability can occur when the ODE is stable and PDE is unstable. S and PU indicate that the system is "always stable" or "possibly unstable", respectively.

	ODE	PDE
(A) Closed-circuit model	S	S
(B) Inhibition from conserved quantities	S	S
(C) Positive feedback	PU	PU

Supplemental Movie S1. An example of the pattern formation by the numerical simulation.
